# Supplementary material for: Quantifying the adaptive landscape of commensal gut bacteria using high-resolution lineage tracking
Source: Nat Commun. 2024 Feb 21;15:1605. doi: 10.1038/s41467-024-45792-0 (PMC10881964; doi:10.1038/s41467-024-45792-0)
Supplement: Supplementary file 1 — Supplementary Information [file 41467_2024_45792_MOESM1_ESM.pdf]

Supplementary Notes

Supplementary Note 1: Effects of excluding lineages

In our description of Eq. (2), we assumed that we could neglect any contributions from the lineages we excluded due to their Tn insertions in "fitness determinant" genes (Methods). To demonstrate that this a good approximation, we show how Eq. (2) can also be derived from a larger model for the full population, which includes both the fitness-determinant genes and other lineages that were excluded by our filtering. To establish notation, we let $\mathcal{L}$ denote the entire set of Tn lineages, while $\mathcal{L}^*$ denotes the subset that passed filtering (Methods). We let $f_{m,\ell}^{(\mathrm{tot})}$ denote the frequency of lineage ℓ in the total population in mouse m, while our previous variable,

$$f_{\ell,m} \equiv \frac{f_{\ell,m}^{(\mathrm{tot})}}{\sum_{\ell \in \mathcal{L}^*} f_{\ell,m}^{(\mathrm{tot})}}\,, \tag{S1}$$

continues to refer to the relative frequency within the subset of included lineages. We assume that the frequency dynamics of the total population can be described by an analogous version of Eq. (2):

$$\frac{\partial f_{\ell,m}^{(\mathrm{tot})}}{\partial t} = [s_{\ell,m}(t) - \overline{X}_m^{(\mathrm{tot})}(t)] f_{\ell,m}^{(\mathrm{tot})} + \sqrt{\Lambda_m^{(\mathrm{tot})}(t) \cdot f_{\ell,m}^{(\mathrm{tot})}} \cdot \eta_{\ell,m}(t)\,, \tag{S2a}$$

where

$$\overline{X}_m^{(\mathrm{tot})}(t) \equiv \sum_{\ell \in \mathcal{L}} s_{\ell,m}(t) \cdot f_{\ell,m}^{(\mathrm{tot})}(t) \tag{S2b}$$

is the mean fitness of the total population, and $\Lambda_m^{(\mathrm{tot})}(t)$ is the total rate of genetic drift. We can recover the dynamics of the renormalized frequencies $f_{\ell,m}$ by taking the time-derivative of Eq. (S1) and substituting Eq. (S2) for $\partial f_{\ell,m}^{(\mathrm{tot})}/\partial t$. Standard manipulations (1) then yield

$$\frac{\partial f_{\ell,m}}{\partial t} = [s_{\ell,m}(t) - \overline{X}_m(t)] f_{\ell,m} + \sqrt{\Lambda_m(t) f_{\ell,m}}\,\eta_{\ell,m}(t) - f_{m,\ell} \sum_{\ell'} \sqrt{\Lambda_m(t) f_{\ell',m}}\,\eta_{\ell',m}(t)\,, \tag{S3a}$$

where $\overline{X}_m(t)$ and $\Lambda_m(t)$ are defined by

$$\overline{X}_m(t) \equiv \sum_{\ell \in \mathcal{L}^*} s_{\ell,m}(t) f_{\ell,m}(t)\,, \quad \Lambda_m(t) \equiv \frac{\Lambda_m^{(\mathrm{tot})}(t)}{\sum_{\ell \in \mathcal{L}^*} f_{\ell,m}^{(\mathrm{tot})}(t)}\,. \tag{S3b}$$

This reduces to the form given in Methods when the the renormalized frequencies ($f_{m,\ell}$) are all small compared to one.

Note that the dependence on the total mean fitness $\overline{X}_m^{(\mathrm{tot})}(t)$ drops out when we focus on the dynamics within $\mathcal{L}^*$. In other words, the relative frequencies of the included lineages only depend on their relative fitnesses with respect to each other. However, in principle, ecological interactions with the excluded lineages (as well as the other species in the community) could still impact the relative fitnesses of the focal lineages; these effects are captured by the time-dependence of $s_{\ell,m}(t)$ and $\Lambda_m(t)$.

Supplementary Note 2: Deriving the moments of lineage frequency

Contribution from within-host lineage dynamics. If the functions $\Lambda_m(t)$, $s_{\ell,m}(t)$, and $\overline{X}_m(t)$ are known, then the dynamics of an individual lineage in Eq. (2) (Methods) can be solved using standard techniques (2). Given an initial frequency $f_{\ell,m}(t_0)$ at time t_0, the moment generating function for the lineage frequency at a later time t is given by

$$H_{\ell,m}(z,t) \equiv \left\langle e^{-zf_{\ell,m}(t)} \right\rangle = \exp\left(-\frac{a_{\ell,m}(t)\cdot z}{1+b_{\ell,m}(t)\cdot z}\right) , \tag{S4a}$$

where the functions $a_{\ell,m}(t)$ and $b_{\ell,m}(t)$ are defined by

$$\begin{aligned} a_{\ell,m}(t) &= f_{\ell,m}(t_0)\cdot e^{\int_{t_0}^{t}(s_{\ell,m}(t')-\overline{X}_m(t'))dt'} , \\ b_{\ell,m}(t) &= e^{\int_{t_0}^{t}(s_{\ell,m}(t')-\overline{X}_m(t'))dt'} \int_{t_0}^{t} dt' \frac{\Lambda_m(t')}{2} e^{-\int_{t_0}^{t'}(s_{\ell,m}(t'')-\overline{X}_m(t''))dt''} . \end{aligned} \tag{S4b}$$

The mean and variance of $f_{\ell,m}(t)$ can then be obtained from the derivatives of $H_{\ell,m}(z,t)$:

$$\begin{aligned} \langle f_{\ell,m}(t)\rangle &\equiv -\left.\frac{\partial \log H_{\ell,m}(z,t)}{\partial z}\right|_{z=0} = a_{\ell,m}(t) , \\ \mathrm{Var}[f_{\ell,m}(t)] &\equiv \left.\frac{\partial^2 \log H_{\ell,m}(z,t)}{\partial z^2}\right|_{z=0} = 2\cdot a_{\ell,m}(t)\cdot b_{\ell,m}(t) . \end{aligned} \tag{S5}$$

Contribution from sequencing noise. Following previous high-resolution lineage tracking studies (2, 3), we assumed that sequencing could be modeled as an additional branching process, with a conditional generating function

$$H^R_{\ell,s}(z|f_{\ell,m_s}(t_s)) \equiv \langle e^{-zR_{\ell,s}} \,|\, f_{\ell,m_s}(t_s)\rangle = \exp\left[-\frac{D_s f_{\ell,m_s}(t_s)\cdot z}{1+(\kappa_s/2)\cdot z}\right] , \tag{S6}$$

where D_s is the total coverage in sample s and κ_s controlling the additional variance beyond Poisson sampling.

By marginalizing over the random value of $f_{\ell,m_s}(t_s)$ using Eq. (S4), we can obtain a corresponding expression for the marginal distribution of the read counts

$$H^R_{\ell,s}(z) = \exp\left[-\frac{a_{\ell,m_s}(t_s)\cdot D_s\cdot z}{1+(\kappa_s/2+b_{\ell,m_s}(t_s)D_s)z}\right] . \tag{S7}$$

Derivatives of the generating function give the mean and variance of read counts quoted in the Methods.

Supplementary Note 3: Distributions of lineage frequency shifts over time

Our initial expectations for the distribution of lineage frequency shifts in Fig. 1H-L were informed by the simplest limit of Eq. (2), in which the vast majority of the focal lineages are effectively neutral ($s_{\ell,m}(t) \approx 0$). For lineages with similar initial frequencies ($f_{\ell,m}(t_0) \approx f_0$), the branching process parameters in Eq. (S4) reduce to a pair of lineage-independent functions,

$$\begin{aligned} a_{\ell,m}(t) &\approx a_m(t) \equiv f_0 \cdot e^{-\int_{t_0}^{t} \overline{X}_m(t')dt'} \,, \\ b_{\ell,m}(t) &\approx b_m(t) \equiv e^{-\int_{t_0}^{t} \overline{X}_m(t')dt'} \int_{t_0}^{t} dt' \frac{\Lambda_m(t')}{2} e^{\int_{t_0}^{t'} \overline{X}(t'')dt''} \,. \end{aligned} \tag{S8}$$

Note that the mean fitness $\overline{X}_m(t)$ can still be non-zero if a small minority of the lineages in the population have non-zero fitness. In this way, the statistical behavior of a large number of neutral marker lineages can in principle provide information about the population parameters $\overline{X}_m(t)$ and $\Lambda_m(t)$ (3).

For example, by substituting Eq. (S8) into Eq. (S7), we see that the average size of a neutral marker lineage declines over time due to competition with fitter lineages in the population ($\overline{X}_m(t) > 0$). Likewise, the variance in the observed read counts grows due to a combination of genetic drift ($\propto \Lambda_m(t)$) and sequencing noise ($\propto \kappa_s/D_s$). However, it can be difficult to apply these heuristics in practice, since the mean and variance can be biased if a small number of highly fit lineages happen to be present in the initial pool. We therefore turned to other characteristics of the lineage frequency distribution that are more robust to small amounts of "contamination" by non-neutral lineages.

For example, in the limit that the lineage fluctuations are small ($\mathrm{Var}(R_{\ell,s}) \lesssim D_s f_0$), one can invert Eq. (S7) to obtain an asympotic expression for the probability distribution of $R_{\ell,s}$ (3),

$$p(R) \propto R^{-3/4} \exp\left[-\frac{(\sqrt{R} - \sqrt{D_s a_m(t)})^2}{\kappa_s/2 + D_s \cdot b_m(t)} \right] \,. \tag{S9}$$

The peak (or mode) of this distribution occurs at a characteristic value R^*, which is given by

$$\frac{R^*}{D_s f_0} = \underbrace{e^{-\int_{t_0}^{t} \overline{X}_m(t')dt'}}_{\substack{\text{competition with} \\ \text{fitter lineages}}} \left[\frac{1}{2} + \sqrt{ \frac{1}{4} - \underbrace{\frac{3\kappa_s}{8 D_s f_0} \cdot e^{\int_{t_0}^{t} \overline{X}_m(t')dt'}}_{\text{sequencing noise}} - \underbrace{\int_{t_0}^{t} \frac{3dt' \Lambda_m(t')}{8 f_0} e^{\int_{t_0}^{t'} \overline{X}_m(t'')dt''}}_{\text{genetic drift}} } \right]^2 \,. \tag{S10}$$

This expression shows that peak of $p(R)$ will decline due to competition with fitter lineages in the population, as well as through genetic drift and sequencing noise. Similarly, Eq. (S9) shows that the characteristic width around this peak will spread out due to genetic drift and sequencing noise. In contrast to the mean and variance above, we expect that these "typical measures" will be robust to the inclusion of a small number of non-neutral lineages in the initial pool. This quantitative picture informed Fig. 1H-L.

Supplementary Note 4: Estimating the overall number of adaptive lineages

Both the rank order curves and inferred distributions of adaptive lineages (Fig. 2 and Supplementary Fig. 6) provide an estimate of the relative numbers of lineages with different relative fitnesses. We note that the interpretation of these distributions is complicated by the fact that we also observed a large degree of negative fitness variation from day 0-4 (Fig. 3A and Supplementary Figs. 6-8), suggesting that there is substantial maladaptive heritable variation in each *Bacteroides* population as well. This makes it difficult to identify the relative fitness that corresponds to the neutral ancestor strain. To be conservative, we therefore only considered a mutation to be adaptive if it increased in frequency over the relevant time interval. This ensures that the lineage is at least as fit as the average fitness of the population.

We also sought to make an independent estimate of the number of adaptive lineages that did not rely on any coarse-graining scheme. First, among the highest N_+ ranked lineages in the discovery cohort, we counted the number of lineages $\hat{n}_+ < N_+$ with consistent (positive) relative fitnesses in the validation cohort. We then compared this to a null expectation for the number of lineages that we would have positively cross-validated by chance, i.e. in the absence of correlations across mice. Under the null hypothesis that lineage fitnesses were uncorrelated across discovery and validation cohorts, we drew N_+ lineages randomly among all ranked lineages, and determined the number $n_{+,0} < N_+$ with positive fitnesses in the validation cohort. The number of lineages with positive relative fitnesses observed in excess over the null model,

$$\Delta n_+ = \hat{n}_+ - n_{+,0} \,, \tag{S11}$$

represents a lower bound on the number of truly adaptive lineages among the first N_+ ranks. To see that this is a lower bound, we define the distribution of true relative fitnesses (in the validation cohort) $\chi_\ell = \int s_\ell(t') - \overline{X}(t')$ in the (ranked) set of lineages as $\rho(\chi)$, and the probability of measuring a positive relative fitness $\hat{\chi} > 0$ in the validation cohort conditioned on χ as $P[\hat{\chi} > 0|\chi]$. Then, the null expectation $\langle n_{+,0} \rangle$ from N_+ draws, $\mathbb{E}[n_{+,0}; N_+]$, can be expressed as

$$\begin{aligned} \mathbb{E}[n_{+,0}; N_+] &= N_+ \big(\int_{-\infty}^{0} d\chi \rho(\chi) P(\hat{\chi} > 0|\chi) + \int_{0}^{\infty} d\chi \rho(\chi) P(\hat{\chi} > 0|\chi) \big) \,, \\ &= N_+ \big(P[\hat{\chi} > 0 \text{ and } \chi < 0] + P[\hat{\chi} > 0 \text{ and } \chi > 0] \big) \,. \end{aligned} \tag{S12}$$

While this empirical null discounts from n_+ the expected number of false positives (the first term), it also discounts the expected number of true positives (the second term). Thus Δn_+ should be thought of as a lower-bound on the number of adaptive lineages. Among HF/HS mice, we estimated at least $\Delta n_+ = 9115 \pm 48$ adaptive lineages in excess of the null expectation among the first 15,000 ranks in *Bc*, 4074±42 out of 10,000 in *Bo*, 2822±45 out of 10,000 in *Bt-VPI*, and 1630±25 out of 4,000 in *Bt-7330*. Similar estimates were obtained for the LF/HPP diet.

Supplementary Note 5: Numbers of adaptive lineages with fitness tradeoffs

We defined a lineage as exhibiting a fitness tradeoff if its relative fitness ($\chi_{\ell,e,t_0:t_1}$) had opposite signs in a pair of environments or time intervals. To robustly detect such lineages, we took a similar cross-validation approach as for single environments (Methods). For example, to detect the fitness tradeoffs between diets over days 4-10, we first used Eq. (7) to estimate $\hat{\chi}_{\ell,H,4:10}$ and $\hat{\chi}_{\ell,L,4:10}$ in discovery cohorts of HF/HS and LF/HPP mice. We used these fitness estimates in the discovery cohort to classify each lineage into one of the four quadrants in the $(\chi_{\ell,H,4:10}, \chi_{\ell,L,4:10})$ plane: the (+,-) and (-,+) quadrants indicate a potential fitness tradeoff, while the (+,+) quadrant indicates a consistent fitness benefit in both environments. We also defined a quantitative measure of the tradeoff magnitude,

$$\hat{T}_\ell = \hat{\chi}_{\ell,H,4:10} \cdot \hat{\chi}_{\ell,L,4:10} \,. \tag{S13}$$

Strong fitness tradeoffs correspond to large, negative values of $\hat{T}_\ell$, while positive values of $\hat{T}_\ell$ indicate consistent fitness benefits. Several example lineages from *Bc* with large values of $|\hat{T}_\ell|$ are illustrated in Fig. 3D-F.

To assess the validity of these measured tradeoffs against noise, we used the validation cohort to check which lineages remained in the same quadrant as their discovery cohort (Supplementary Fig. 10). To control for multiple hypothesis testing, we then compared the observed number of lineages found in the same quadrant to the following null expectation. If we designated N_L lineages as putatively fit in L and unfit in H based on their quadrant in the discovery cohort, we drew N_L null lineages without replacement among those with positive L fitness in the discovery cohort. We then determined how many of these N_L null lineages were found in the consistent quadrant in the validation cohort. This null model preserves the correlations in positive fitness across mice fed one diet, and assumed that measured tradeoffs in the other diet were simply due to biological or technical noise. We performed an analogous comparison to validate lineages that were putatively fit in H and unfit in L, drawing null lineages with positive H fitness in the discovery cohort. The excess of lineages with consistent behavior across validation and cohorts over the null expectation in both categories indicated a statistical enrichment for genuine fitness tradeoffs in hundreds of lineages (Supplementary Fig. 10). We used an analogous approach to identify “generalist” lineages with consistent fitness benefits in both environments, and observed a similar enrichment of these mutations as well (Supplementary Fig. 10).

Supplementary Note 6: Identifying clusters of phenotypically similar lineages

A key limitation of lineage tracking methods is that they do not provide direct information about the genetic targets of adaptation. This limitation can in principle be overcome by performing whole-genome sequencing on isolated adaptive lineages (4–6). Supplementary Fig. 13 shows, for each *Bacteroides* library and timepoint, the total number of sampled isolates necessary to recover a given number of adaptive lineages. This highlights an efficient sampling window, over days 4-10, where adaptive lineages are diverse and abundant in the library, such that $> 10\%$ of sampled isolates represent different adaptive lineages. In addition, multiple isolates per adaptive lineage may be required to rule out neutral or deleterious hitchhiking mutations (acquired *in vitro* or during isolation) that may be especially prevalent in *Bacteroides*, which harbor many genomic loci that rapidly mutate (e.g. by invertible promoters) to generate phenotypic "phase variation" (7, 8). This requirement more strongly favors later sampling intervals, where fewer adaptive lineages dominate a population (Supplementary Fig. 13).

However, isolating and sequencing the required number of lineages of a particular population can be especially challenging in multi-species settings like Fig. 1A, where a whole population may reside at low frequencies. This could be overcome by *in vitro* enrichment of a population of interest by taxon- or strain-specifc culturing (9–11), or by cultivation-free methods using droplet microfluidics (12). Other emerging technologies enable selective isolation of lineages from barcoded populations, but require post-hoc design of lineage-specific probes (e.g. CRISPR-induced reporters; 13). The functional impact of individual mutations is also typically only understood by grouping them into larger functional units (4, 14), which depends on the quality of genome annotation and can be sensitive to the (ad hoc) degree of coarse-graining.

In light of the above challenges, we propose a different approach for annotating mutations that bypasses the intermediate sequencing step, which makes it particularly well-suited for analyzing large numbers of adaptive lineages. This approach is motivated by recent work in functional genomics (15) and experimental evolution (16, 17) showing that the function of genomic variants can often be inferred by examining their pleiotropic tradeoffs across large numbers of *in vitro* environments.

The premise of this approach is that two functionally distinct mutations can have similar fitness effects in one environment, but their fitness effects in other environments may expose differences in their underlying molecular phenotypes. An example of this behavior is shown in Fig. 3: the lineages in panels E and F had similar relative fitnesses in both the HF/HS and LF/HPP diets between days 4-10, but their relative fitnesses diverged between days 0-4. These consistent differences allow us to conclude that the adaptive lineages were likely driven by different underlying mutations.

By the same logic, if two adaptive lineages exhibit the same growth rate differences across a sufficiently large number of environmental conditions, then it is likely that they have acquired either the same mutation or a pair of functionally similar variants (e.g. two mutations in the same gene, or perturbations of a common pathway or module). If the genetic basis of one of these lineages can be determined (e.g. via whole-genome sequencing), then this information can serve as a "functional annotation" for the other lineages in the same phenotypic cluster. Even in the absence of additional sequencing information, the presence of phenotypically similar lineages can provide important information about the degree of evolutionary parallelism within a

population (18), and the number of independent phenotypes that can be tuned by natural selection (17). The resulting clusters can also be annotated directly based on the environments in which they exhibit strong fitness effects. Previous work has used this approach to infer the function of poorly annotated genes in diverse species of bacteria (15, 19), by comparing the “fitness profiles" of gene knockouts obtained from genome-wide Tn-Seq screens across a diverse range of environments. Here we show how extensions of this approach can be used to infer the functional targets of adaptation for some of the secondary mutations in Figs. 1-3.

Supplementary Note 7: Estimating genetic drift *in vivo* when selection is widespread

Our results in the main text show that positive selection is a pervasive force during *in vivo* colonization (Figs. 1-3). The opposing force of genetic drift could also play an important role, reflecting enhanced spatial structure within the gut and/or transient population bottlenecks during engraftment.

In the absence of natural selection [$s_\ell(t) = \overline{X}(t) = 0$], genetic drift leaves a well-known signature in the variance of a lineage's frequency,

$$\mathrm{Var}(\hat{f}_{\ell,m}(t)) = \langle \hat{f}_{\ell,m}(t) \rangle \cdot \left(\frac{\kappa_t}{D_t} + \int_0^t dt' \Lambda_m(t') \right) = f_{\ell,m}(0) \cdot \left(\frac{\kappa_t}{D_t} + \int_0^t dt' \Lambda_m(t') \right) , \tag{S14}$$

which follows from the model in Eq. (6a). This simple behavior underlies several common methods for inferring the strength of genetic drift from frequency trajectory data (20–24). In the presence of natural selection, this behavior takes on a more complicated form,

$$\mathrm{Var}(\hat{f}_{\ell,m}(t)) = \langle \hat{f}_{\ell,m}(t) \rangle \cdot \frac{\kappa_t}{D_t} + \langle \hat{f}_{\ell,m}(t) \rangle^2 \int_0^t \frac{dt' \Lambda_m(t')}{f_{\ell,m}(0)} e^{-\int_0^{t'} [s_{\ell,m}(t'') - \overline{X}_m(t')] dt''} , \tag{S15}$$

which depends on the fitness of the focal lineage ($s_\ell(t)$) as well as the mean fitness of the population ($\overline{X}(t)$). Estimating the strength genetic drift in this more general scenario is more challenging.

Previous work has shown that the strength of genetic drift can be inferred from the trajectories of a collection of neutral marker lineages ($s_\ell(t) = 0$) with a sufficiently dense time series (2, 3). However, this approach suffers from two key limitations that make it difficult to apply in the present case. First, if a substantial fraction of the marker lineages have non-neutral fitnesses, this method will be strongly biased by the variation in fitness among marker lineages, and tend to overestimate the strength of genetic drift. Second, this approach also requires multiple sequencing replicates at each timepoint to distinguish the contributions of genetic drift from technical noise (3, 25).

We sought to exploit a different feature of Eq. (S15) to infer the strength of genetic drift when natural selection is sufficiently widespread. Our approach is based on the observation that contribution from technical noise depends on the present-day frequency of the lineage, while the contribution from genetic drift depends on the historical trajectory of the lineage as well. For a given present-day frequency, a higher fitness lineage must have been present at a lower frequency in the initial timepoint, and will therefore have experienced stronger genetic drift on the way to its present-day frequency. This suggests that the strength of genetic drift will show up as a systematic correlation between $\mathrm{Var}(\hat{f}_{\ell,m}(t))$ and $\hat{f}_{\ell,m}(0)$.

To make this intuition more precise, we focused on a simple version of the model in Eq. (2), in which the relative fitnesses and strength of genetic drift are approximately constant over the relevant time interval. In particular, for every mouse m with host environment e, we let $\chi_{\ell,e}(t) \approx \chi_{\ell,e}$ and define $\Lambda_m(t) \simeq 1/N_e\tau_e$, where N_e is the effective population size and τ_e is the effective generation time in the gut. In this limit, Eq. (S15) reduces to

$$\mathrm{Var}(\hat{f}_{\ell,m}(t)) \approx \frac{\kappa_{m,t}}{D_{m,t}} \cdot \langle \hat{f}_{\ell,m}(t) \rangle + \frac{t}{N_e \tau_e} \cdot \frac{\langle \hat{f}_{\ell,m}(t) \rangle \left[\langle \hat{f}_{\ell,m}(t) \rangle / f_\ell(0) - 1 \right]}{\log \left[\langle \hat{f}_{\ell,m}(t) \rangle / f_\ell(0) \right]} , \tag{S16}$$

where

$$\langle \hat{f}_{\ell,m}(t) \rangle = f_\ell(0) e^{\chi_{\ell,e} \cdot t} . \tag{S17}$$

The expectation in Eq. (22) follows by substituting Eqs. (S16) and (S17) into Eq. (21).

Comparison to an existing method. We compared our estimates to STAMPR (26), which also estimates genetic bottlenecks from lineage-tracking data. In contrast to our approach, STAMPR assumes that the majority of lineages are effectively neutral, and that technical noise is effectively Poisson ($\kappa_t = 1$). When applying this approach to our data, we find that STAMPR yields typical estimates of $N_e \tau_e$ that range from $\sim 10^5$ to $\sim 10^{5.5}$ days, at least an order of magnitude smaller than our own estimates (Fig. 5E). Thus, our approach makes comparatively conservative estimates of population bottlenecks compared to the state of the art.

Limitations of our approach. However, these initial estimates should be treated with a degree of caution, since our simple regression model made a number of simplifying assumptions that may not hold in practice. Chief among these was the assumption that $\Lambda(t)$ and $\chi_\ell(t)$ are approximately constant over the relevant time interval. Residual noise could also pose problems for the regression step, though we have tried to mitigate these issues by averaging many lineages within bins. The reasonable performance of our algorithm on simulated data suggests that these issues have a limited impact in the parameter ranges we examined here. Further refinements to our algorithm – and extensions to other sources of biological noise – remains an interesting topic for future work.

Supplementary Figures

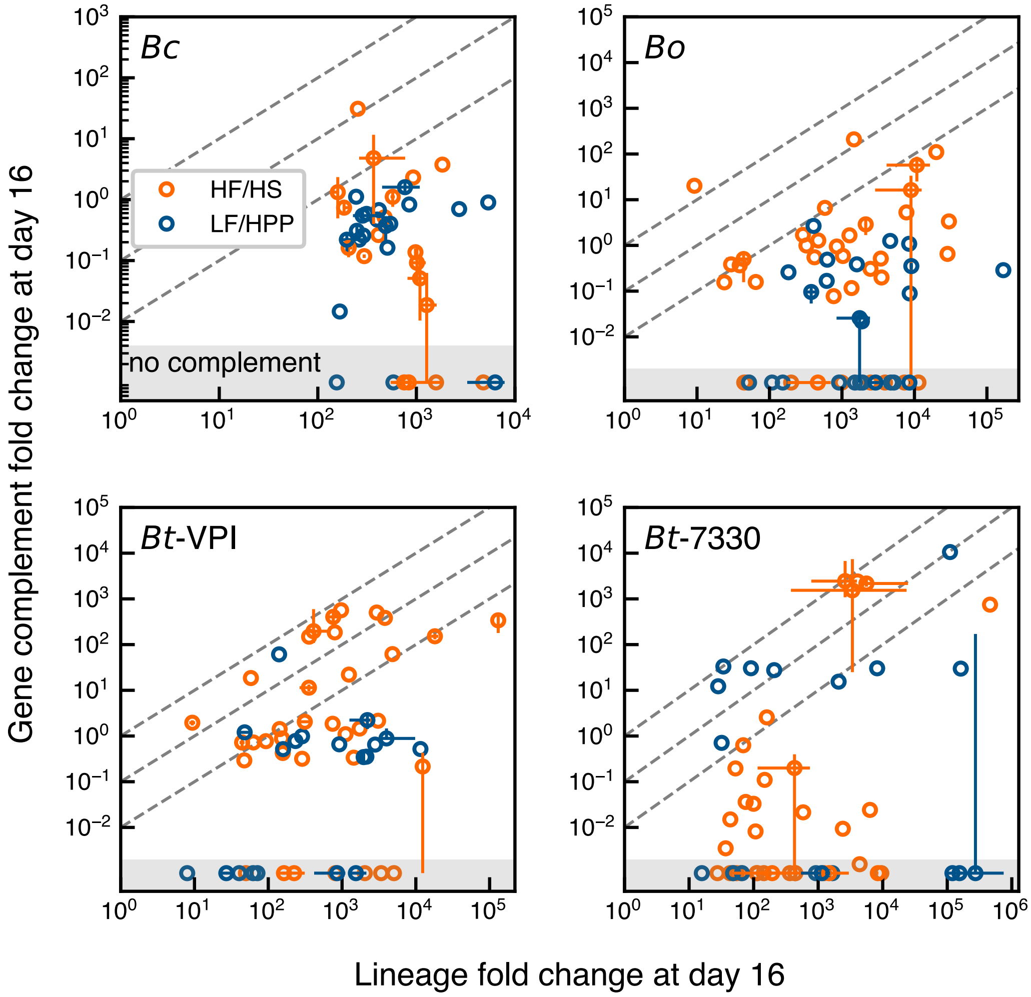

Supplementary Figure 1: Most lineages that reached intermediate frequency by day 16 were not driven by beneficial Tn insertions. For each mouse in the HF/HS (red, $n = 5$) or LF/HPP (blue, $n = 3$) diets, the fold change of the top 10 largest lineages at day 16 is plotted against the fold change of other Tn lineages in the same gene (the gene complement, Methods). Lineages among the top 10 in multiple mice in the same diet are represented by their median (circles) and minimum and maximum values (lines) across those mice (lines). Lineages in the grey region either have Tn insertions in intergenic regions or gene complements measured at zero frequency at day 16.

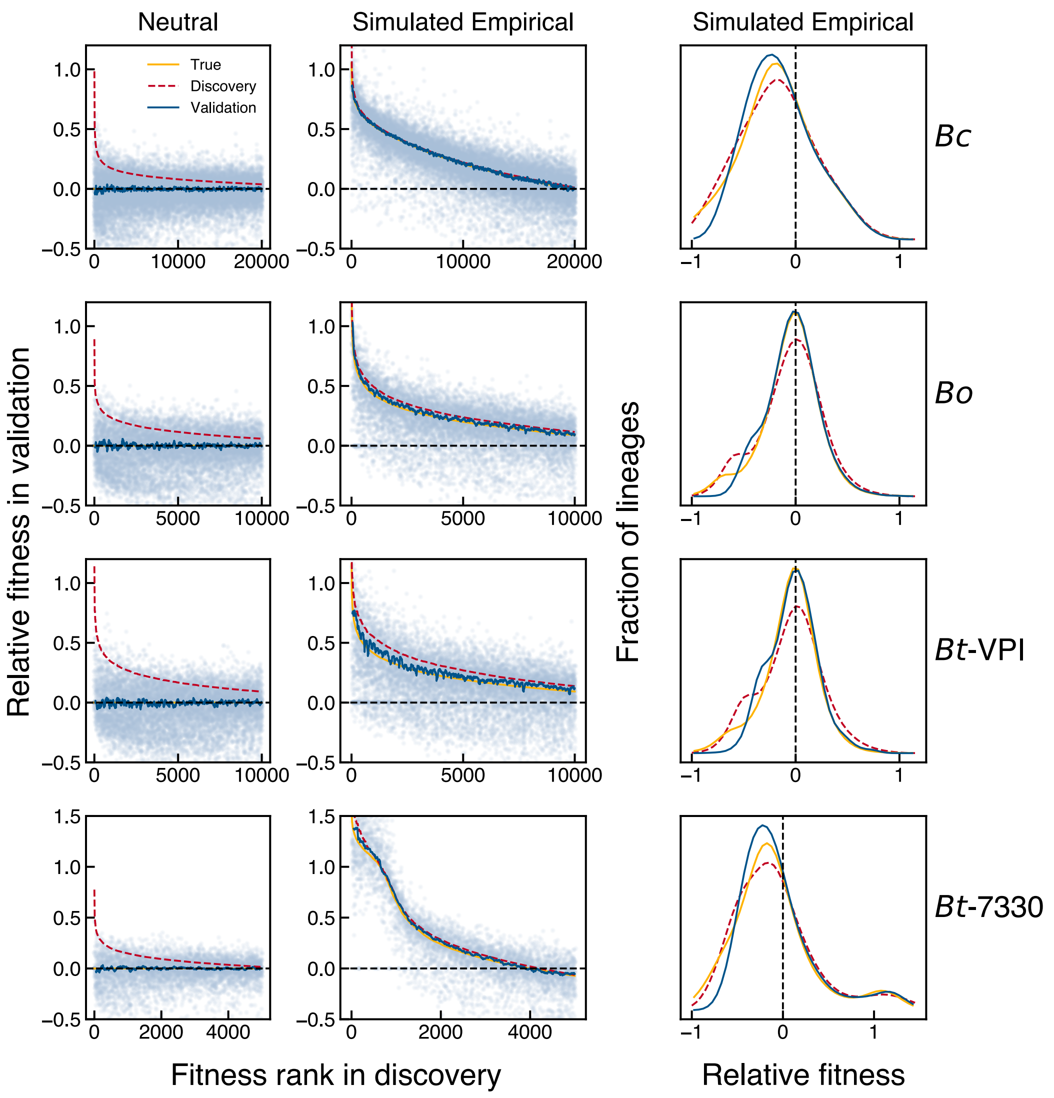

Supplementary Figure 2: Cross-validation consistently estimates relative fitnesses of lineages in simulated populations. For each *Bacteroides* library (row), 9 populations were initialized with the distribution of lineage frequencies estimated from the pooled day 0 input libraries. In one set of simulations (left column), every lineage's fitness was set to 0 to mimic a neutral scenario. In another set (center column), each lineage's fitness was set to its average across 9 HF-fed mice during days 0-4, according to Eq. (7). Lineage dynamics were simulated as described in the Methods, and then filtered, ranked, and plotted using the same procedures as Fig. 2. The right plot compares the true distribution of lineage relative fitnesses to that estimated by inverting the coarse-grained rank-order curve in the center plot (Methods).

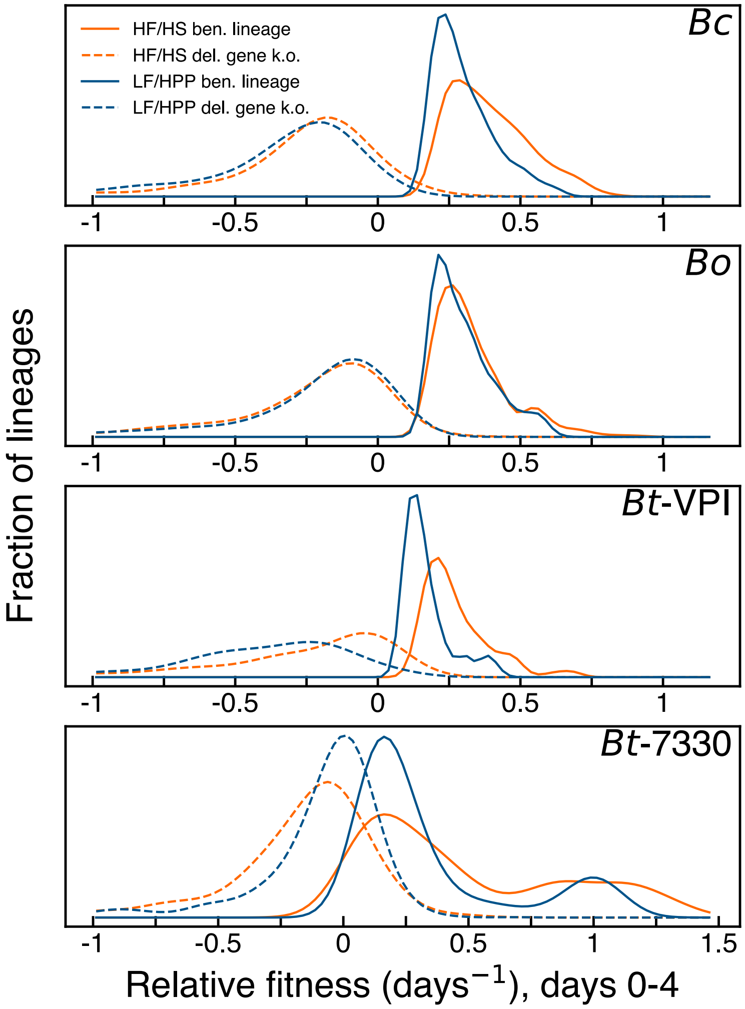

Supplementary Figure 3: Relative fitnesses of adaptive lineages compared to "fitness determinant" gene knockouts identified in previous work. Distribution of cross-validated relative fitnesses (n=4 HF/HS and 3 LF/HPP validation mice, respectively) of the fittest 10,000 (*Bc*), 5,000 (*Bo* and *Bt-VPI*), or 3,000 (*Bt-7330*) lineages, which were estimated with a separate discovery cohort of mice (n=5 HF/HS and 4 LF/HPP mice). These were compared to the relative fitnesses (estimated in the same validation mice) of deleterious gene knockouts annotated by Wu *et al.* (27). Frequencies of gene-knockouts were estimated by summing all reads from Tn insertions falling in the gene, as defined in the Methods. Distributions were smoothed with Gaussian kernel density estimation, as described in the Methods.

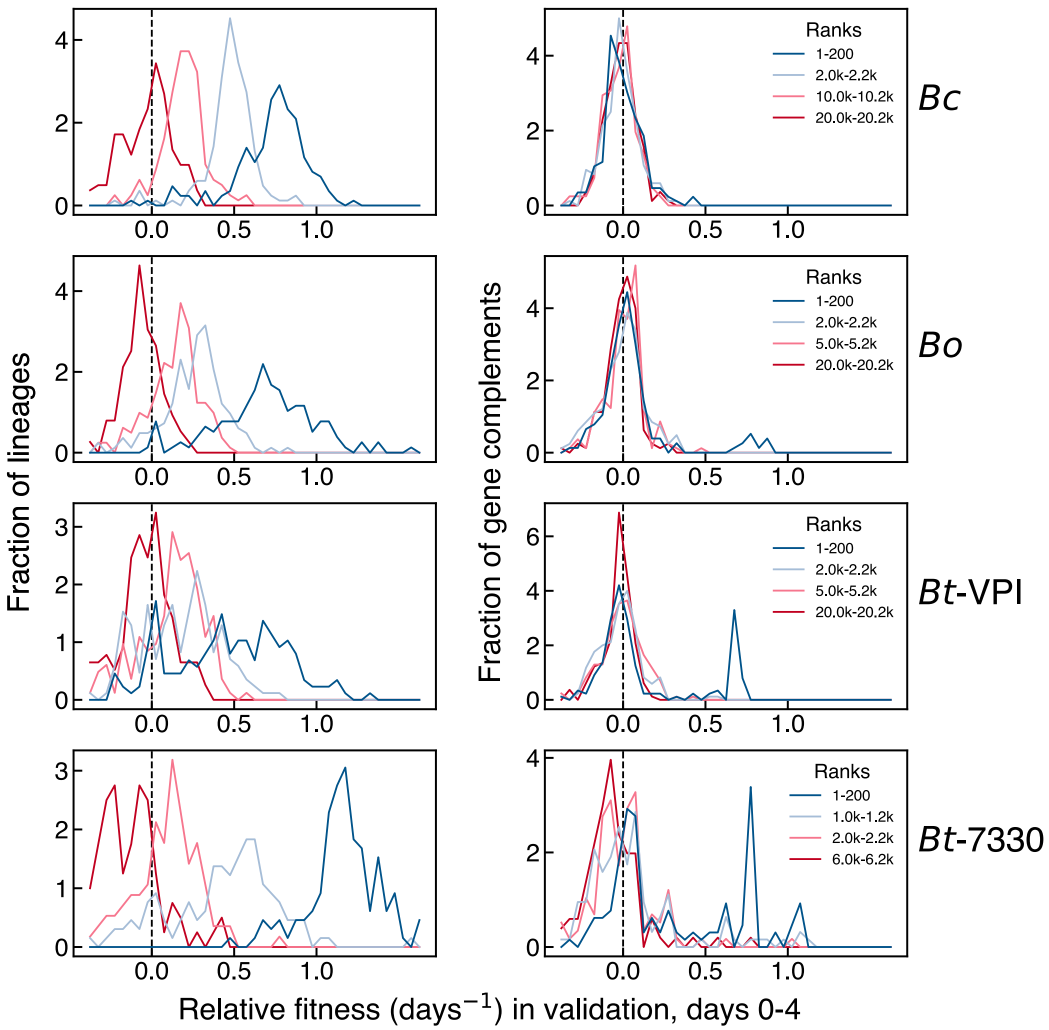

Supplementary Figure 4: Conditional distributions of relative fitnesses at different ranks. In each *Bacteroides* library (**row**), 4 sets of lineages were collected, spanning different ranks of relative fitness during days 0-4 in the HF/HS discovery cohort. For each set, the distributions of relative fitnesses of the lineages (**left**) and their gene complements (**right**, Methods) in the HF/HS validation cohort during days 0-4 are plotted. The same cohorts of HF/HS discovery and validation mice were used as in Fig. 2 and Fig. 5. In each of the 4 *Bacteroides* libraries, the mode of adaptive lineages' relative fitness remains positive over thousands of ranks, suggesting positive fitness variation across thousands of lineages. Conversely, the mode of their respective gene complements' fitnesses are centered at zero relative fitness, independent of rank, suggesting that most lineages' fitness benefits do not derive from the gene-knockout effects of their original Tn insertions.

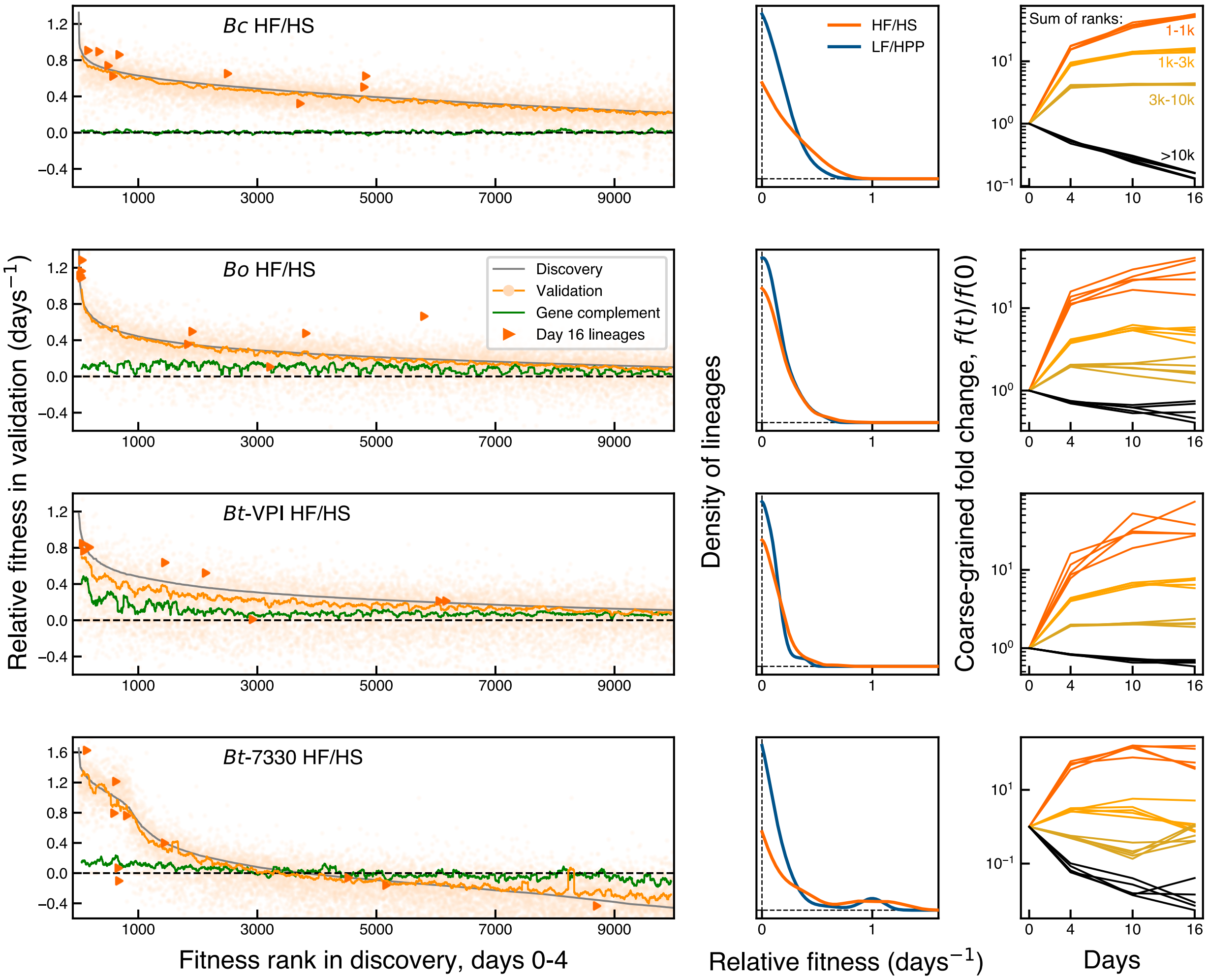

Supplementary Figure 5: Analogous version of Fig. 2C-H for all four species. The same sets of discovery (n=5) and validation (n=4) mice are used as in Fig. 2. To emphasize differences in the numbers and fitnesses of adaptive lineages, the first 10,000 ranks are shown for all 4 *Bacteroides* species.

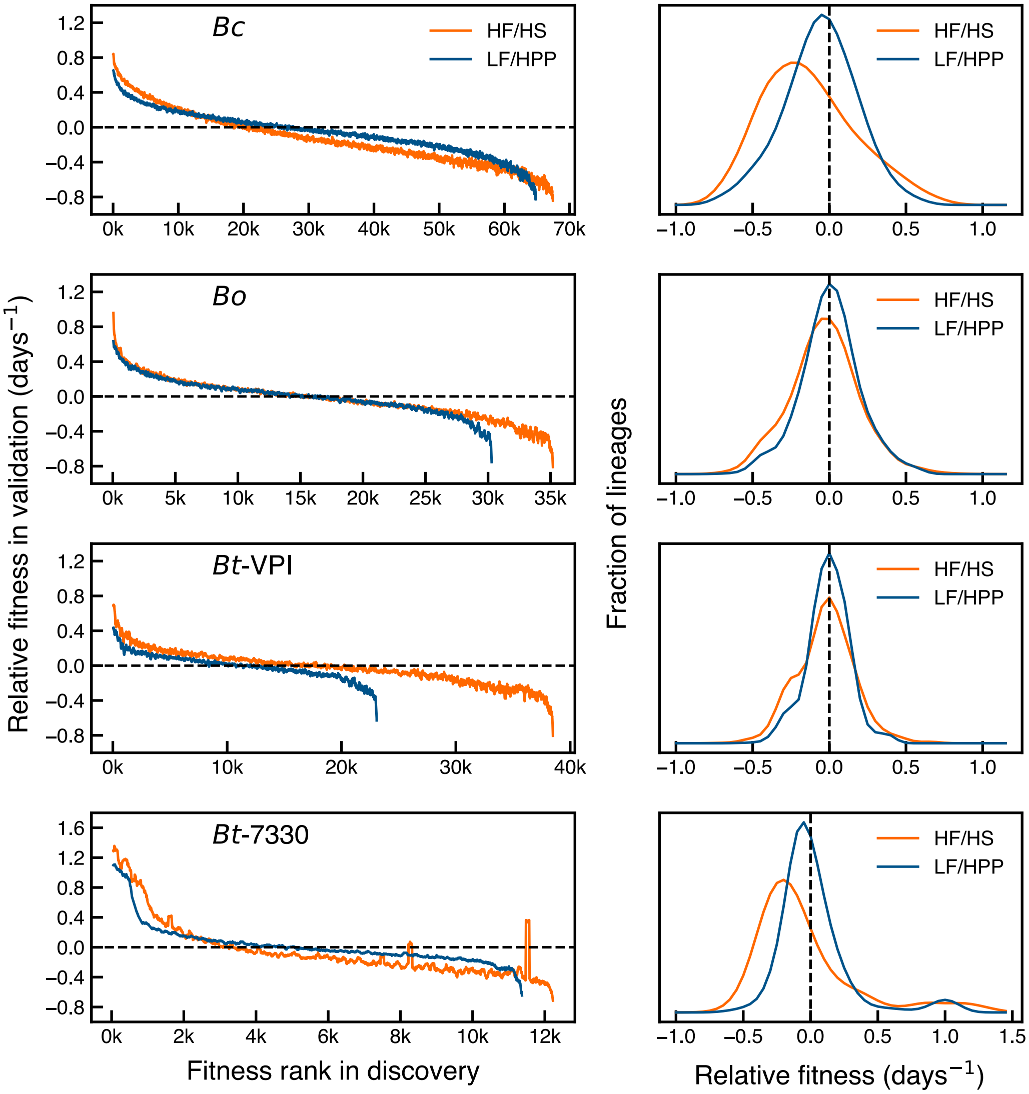

Supplementary Figure 6: Full rank-order curves and distribution of relative fitnesses in the HF/HS and LF/HPP diets. For the HF/HS diet, n_D=5 mice were used for discovery and n_V=4 mice were used for validation, while in the LF/HPP diet, n_D=4 and n_V=3. Different sets (and numbers) of lineages pass the filtering steps (Methods) in each diet.

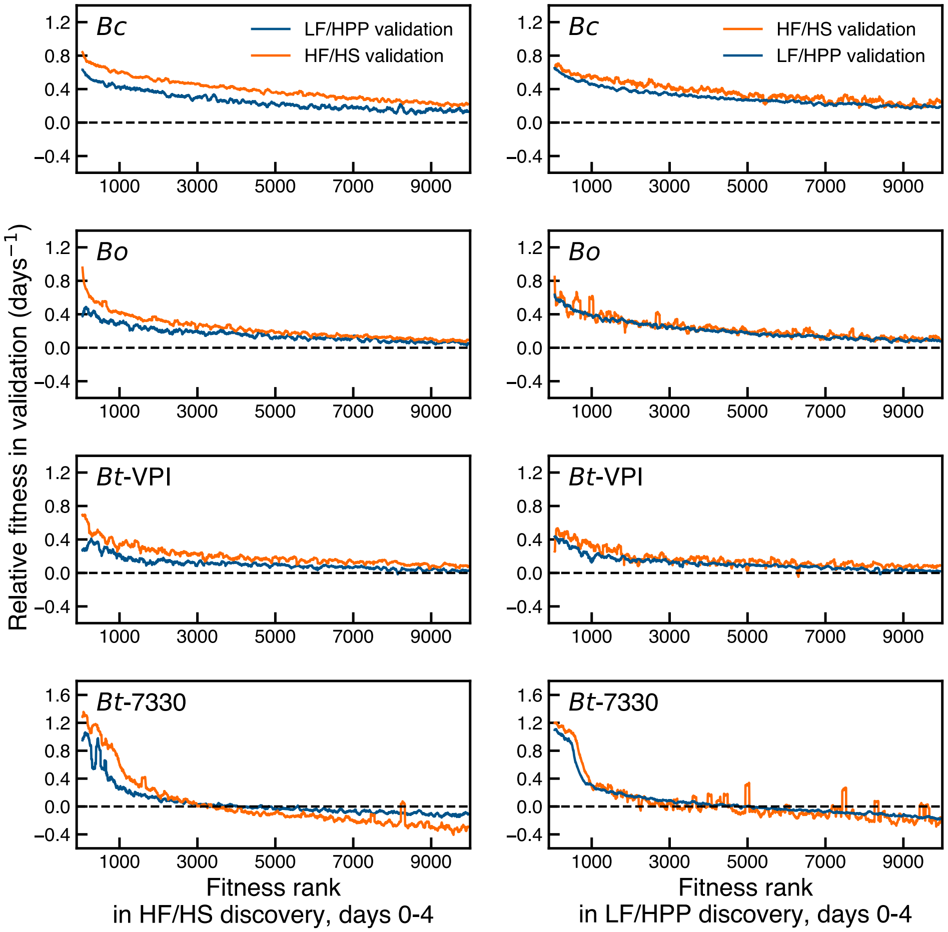

Supplementary Figure 7: Lineage fitnesses are strongly correlated across diets during days 0-4. Relative fitnesses during days 0-4 in the HF/HS and LF/HPP diets plotted against rank order of relative fitnesses during days 0-4 in a discovery cohort with the same or different diet. Each curve is a running coarse-grained average of 100 lineages.

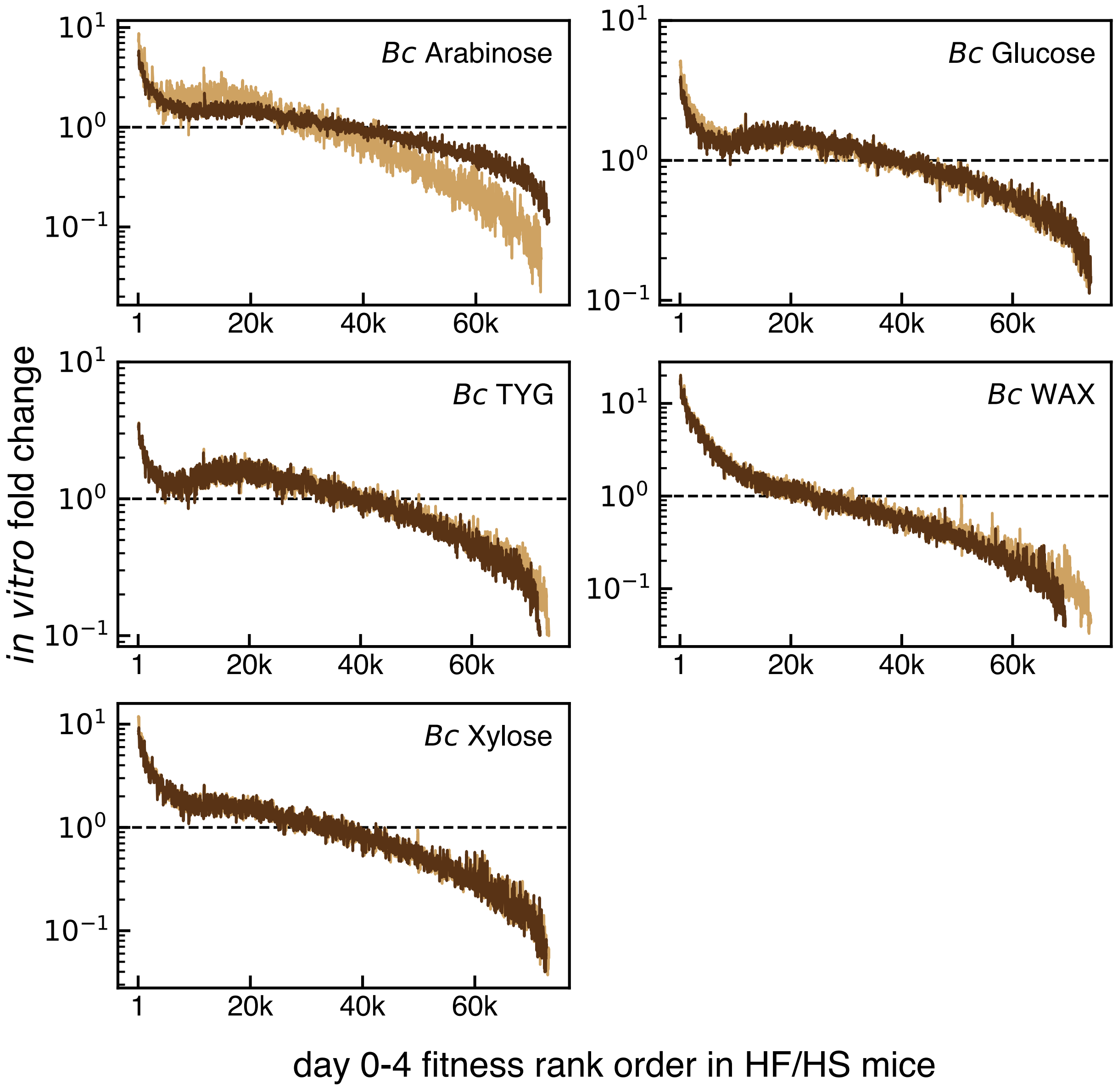

Supplementary Figure 8: *In vivo* relative fitnesses correlate with *in vitro* relative fitnesses for a subset of media in *Bc*. Fold changes of *Bc* lineages in overnight cultures of different media, ranked by their relative fitnesses during days 0-4 in HF/HS mice. The curves represent running averages of 100 ranked lineages (Methods). For each medium, two independent cultures (gold, brown) demonstrate the reproducibility of the relative fitnesses of coarse-grained lineages.

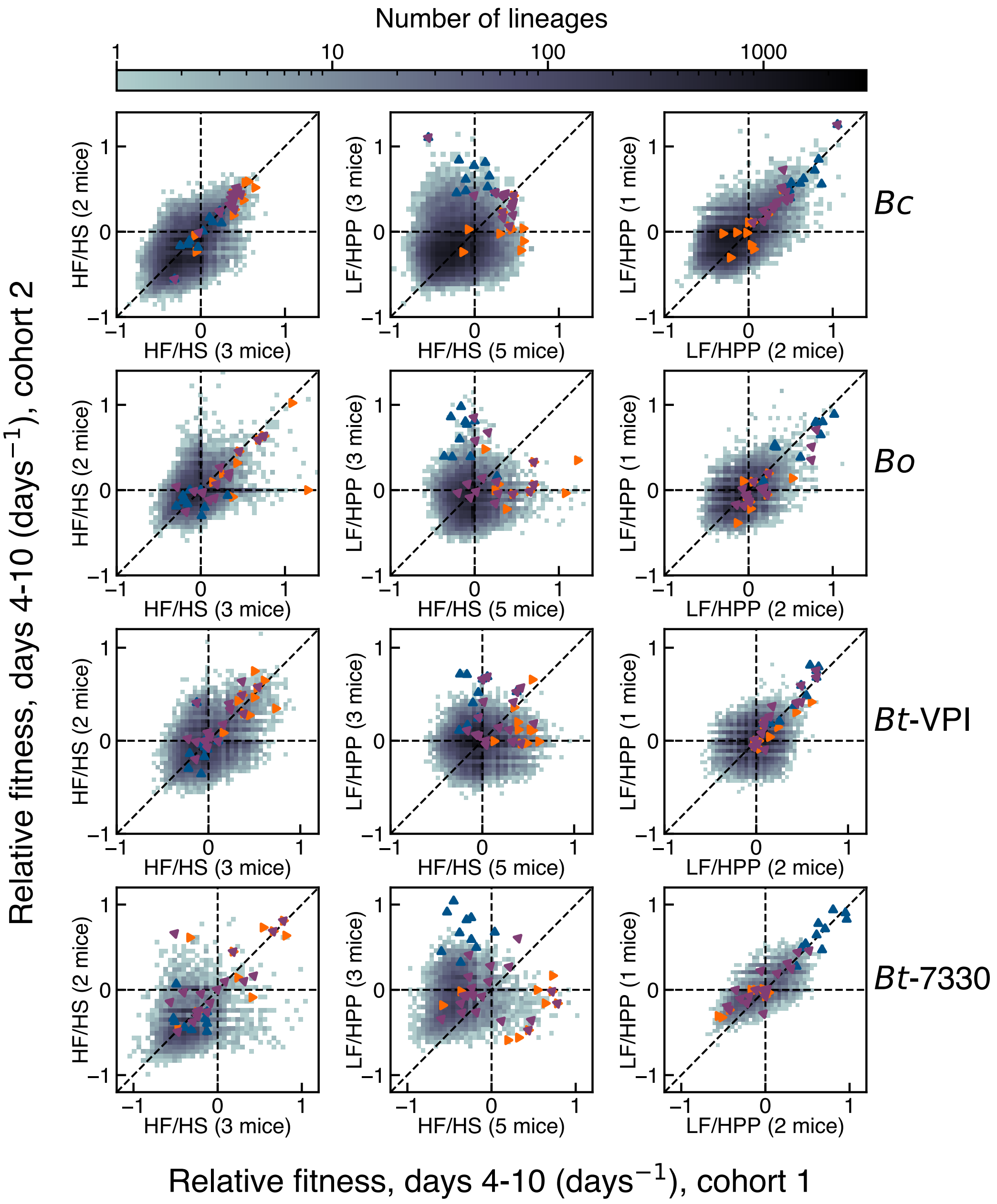

Supplementary Figure 9: Lineage fitnesses during days 4-10 are correlated within but not between diets. For each panel, average relative fitnesses during days 4-10 were calculated in nonoverlapping sets of HF/HS and LF/HPP mice (Methods). Rows correspond to different *Bacteroides* species and columns to the same sets of mice. Triangles are the 10 largest lineages at day 16 in the HF/HS (orange), LF/HPP (blue), or alternating (purple) diets.

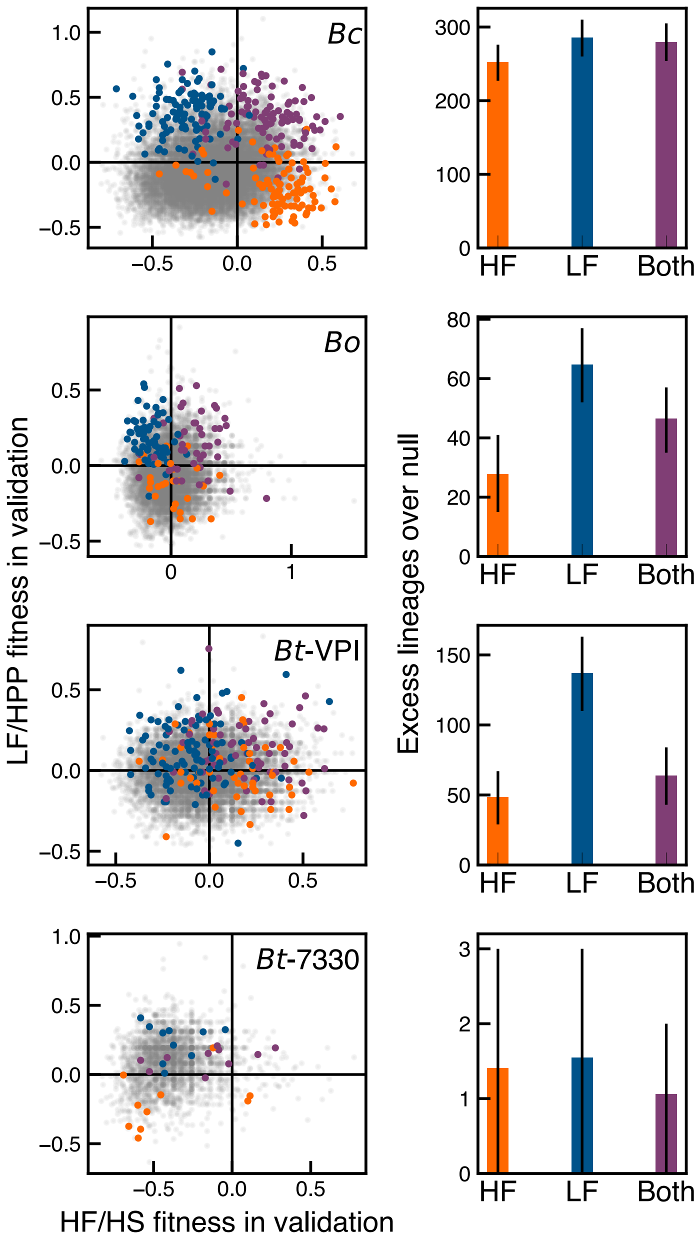

Supplementary Figure 10: Hundreds of lineages exhibit relative fitnesses that depend on diet at later times. For each *Bacteroides* species, the left panel shows the joint distribution of relative fitnesses between days 4-10 (grey) in the a cohort of 2 HF/HS and 1 LF/HPP mice. Highlighted lineages have the largest value of the tradeoff statistic $|T_\ell|$ in a separate discovery cohort of 3 HF/HS and 2 LF/HPP mice (Supplementary Note 5). The right panel shows the excess number of lineages, over a null expectation (Supplementary Note 5), in each quadrant in the discovery cohort that fell in the same quadrant in the validation cohort (dark shading). Error bars indicate 95% confidence intervals in the excess lineages, from repeated draws of the null distribution.

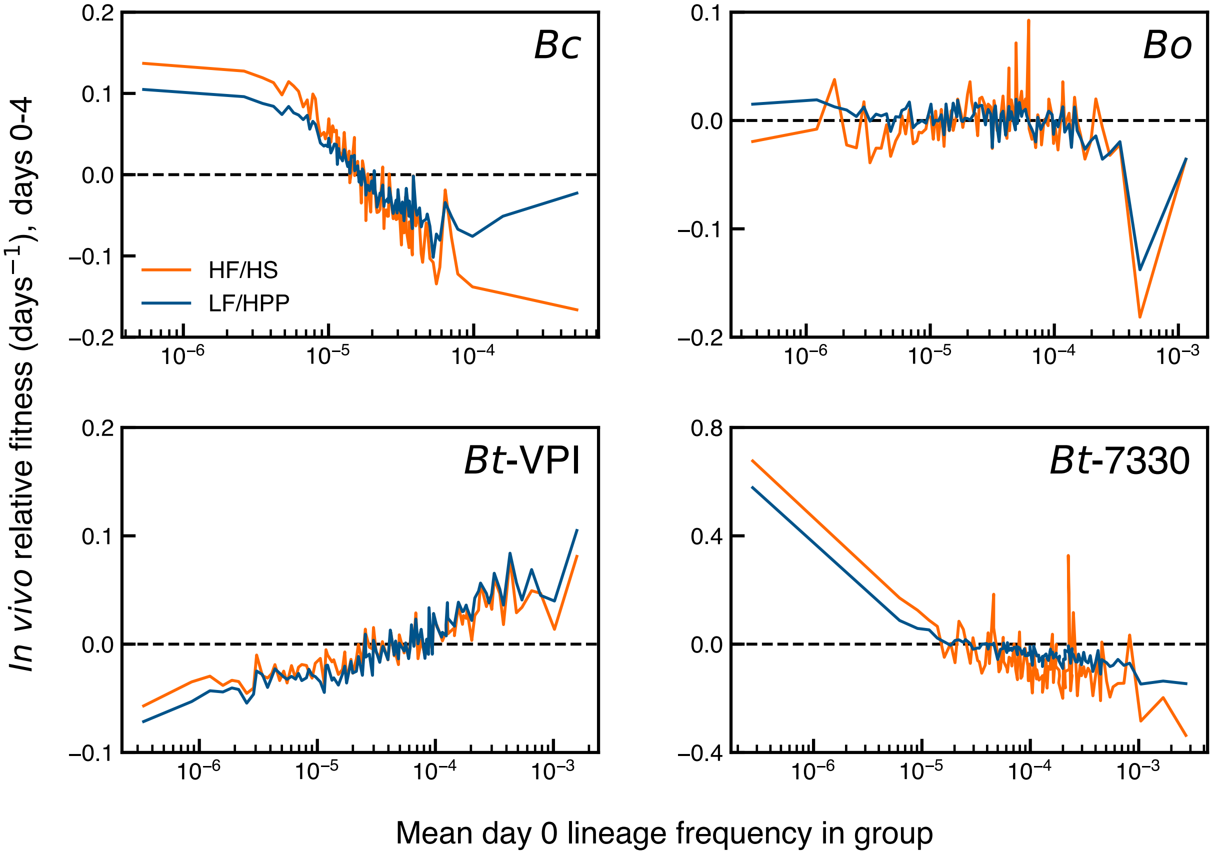

Supplementary Figure 11: Lineage fitnesses are anti-correlated with their frequency in the input library in *Bc* and *Bt-7330*. For each species, day 0 lineage frequencies were estimated and ranked in a discovery pool of input libraries. Lineages were then coarse-grained into 100 super-lineages of roughly equal day 0 frequency $\sim 1\%$ using the procedure described in the Methods. The relative fitnesses of these super-lineages were estimated during days 0-4 *in vivo* using a separate validation pool of input libraries to re-measure initial frequencies. Relative fitnesses in HF/HS (orange) and LF/HPP (blue) cohorts of mice are plotted against the average lineage frequency within each super-lineage, measured in the validation input pool. *Bc* and *Bt-7330* exhibit strong anti-correlations between *in vivo* fitness and initial frequency, whereas *Bt-VPI* shows the opposite trend.

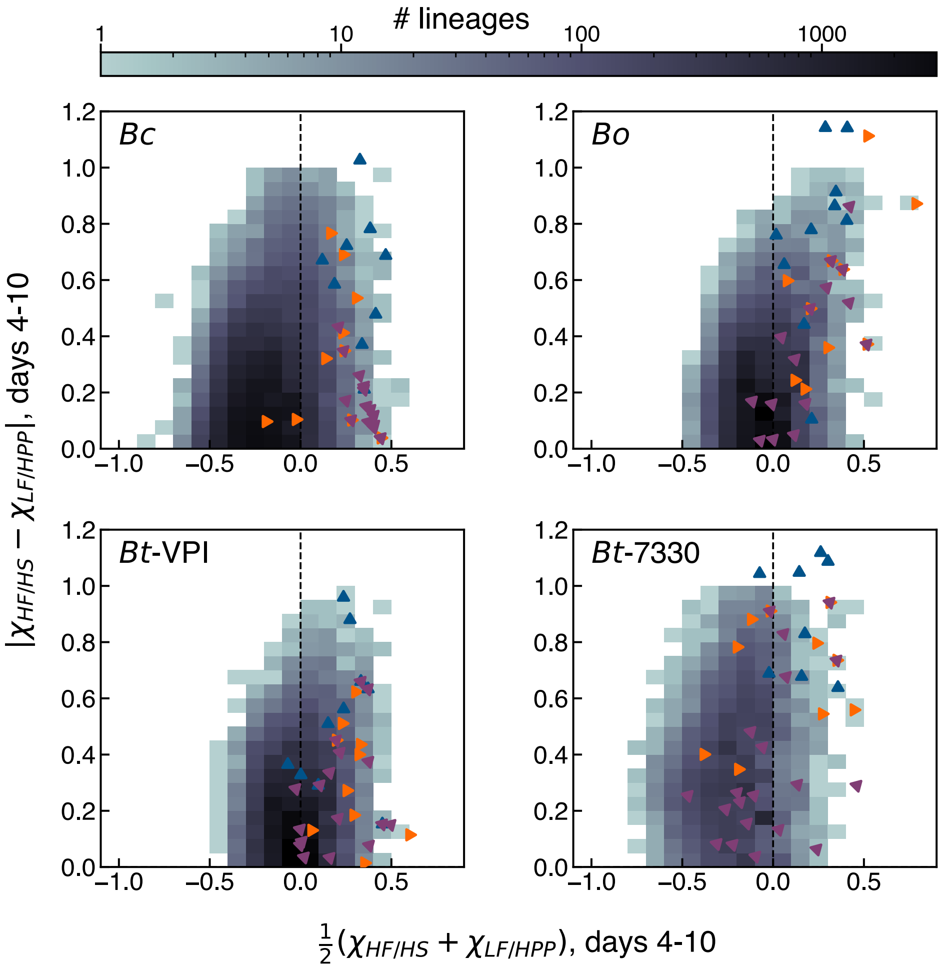

Supplementary Figure 12: Analogous version of of Fig. 3C computed for diet-averaged fitnesses. For each of the lineages in Fig. 3C, we computed a diet-averaged fitness $\chi_{\text{avg}} = (\hat{\chi}_{\ell,H,4:10} + \hat{\chi}_{\ell,L,4:10})/2$ and the corresponding off-diagonal component $|\hat{\chi}_{\ell,H,4:10} - \hat{\chi}_{\ell,L,4:10}|$ between days 4-10. An analogous calculation was carried for the other three species. This projection shows that the largest lineages in the alternating diets (purple triangles) had higher diet-averaged fitness and smaller tradeoffs in *Bc*, despite their smaller representation in the underlying distribution.

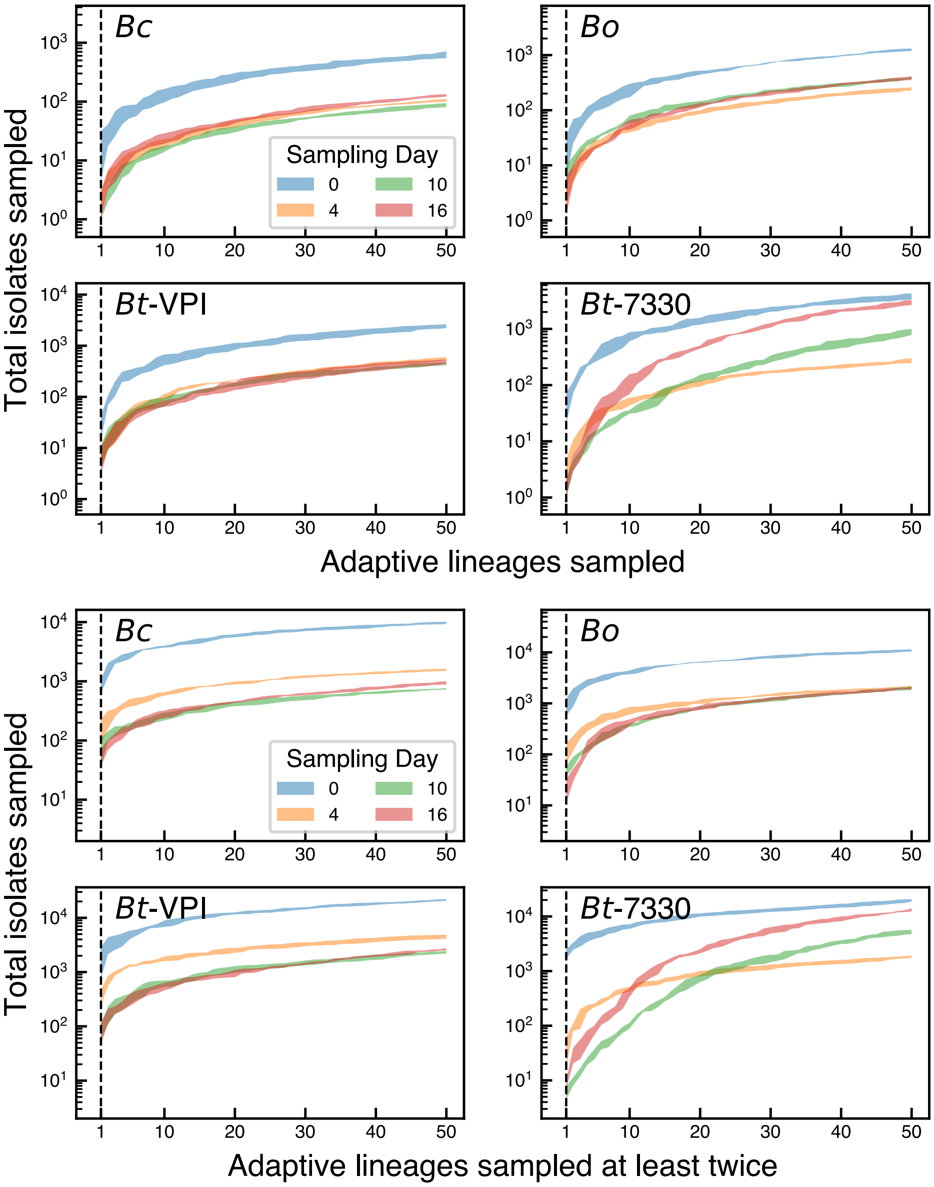

Supplementary Figure 13: Number of isolates required to sample the adaptive diversity in each library. **Top**: the total number of isolates from a library required to sample a given number of adaptive lineages at least once. Adaptive lineages were defined as the highest 10000 (*Bc*), 5000 (*Bo* and *Bt-VPI*), and 2000 (*Bt-7330*) HF/HS fitness ranks, as represented in Fig. 5. Lineage frequencies measured in a single representative HF/HS mouse were used. Different colors represent different sampling days, and shaded regions represent interquartile ranges. **Bottom**: the total number of isolates required to sample a given number of adaptive lineages, with each such lineage sampled at least twice.

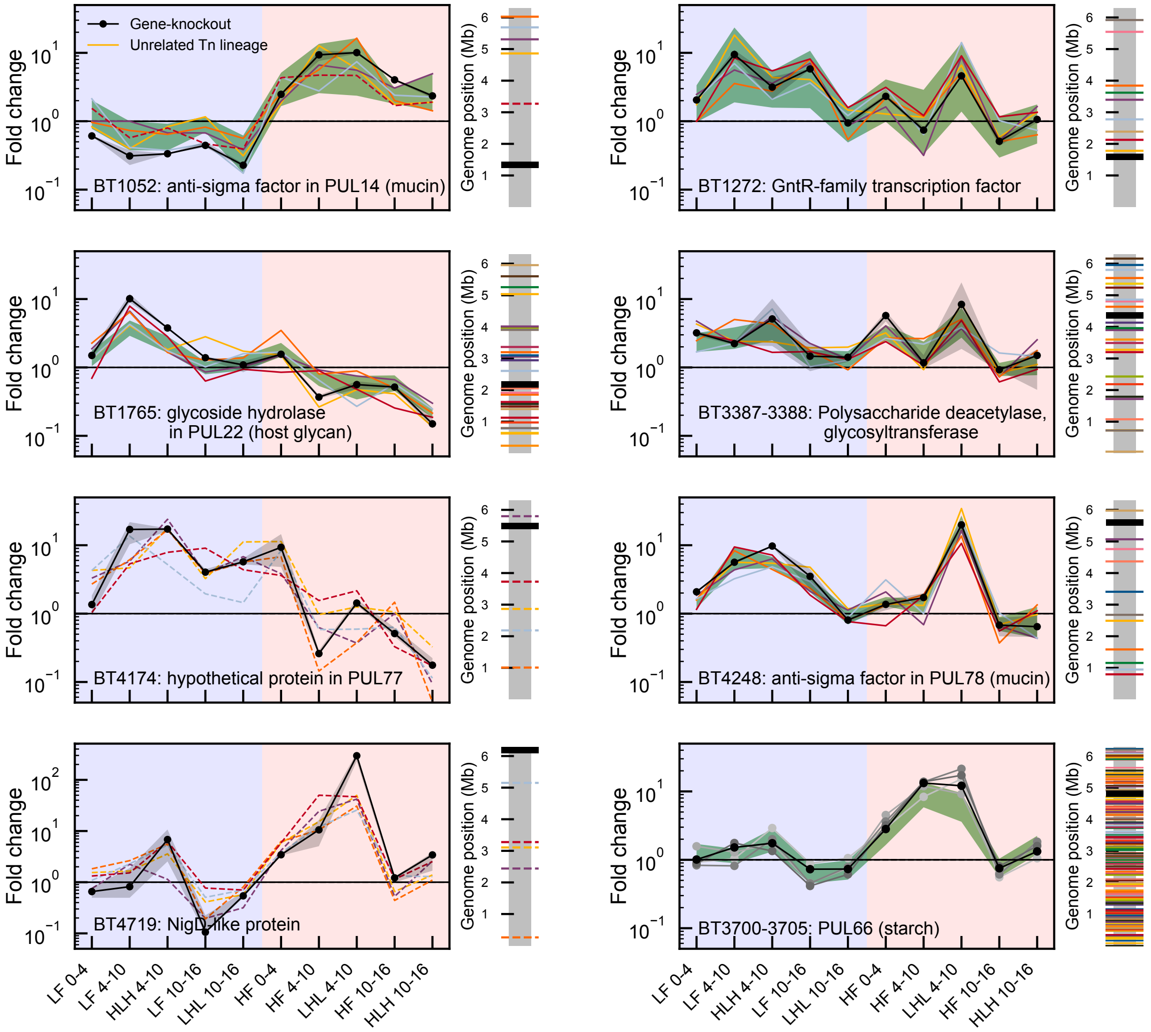

Supplementary Figure 14: Additional examples of knockout-like lineages in *Bt-VPI*. Analogous versions of Fig. 4B for eight additional example genes (black) where unrelated Tn lineages exhibit similar fitness tradeoffs (colored curves; Methods). For clusters with at least (fewer) than 10 lineages, green bands are IQR (full range). For gene knockouts with fewer than 5 unrelated Tn lineage within a fitness profile distance $d < d^* = 2$ (Methods), the 5 lineages with the smallest distances are plotted; dashed lines denote lineages with $d > 2$. At bottom right, 235 Tn lineages cluster with gene knockouts of *BT3700-BT3705* (excluding *BT3704*) sharing highly similar fitness profiles (shades of grey and black).

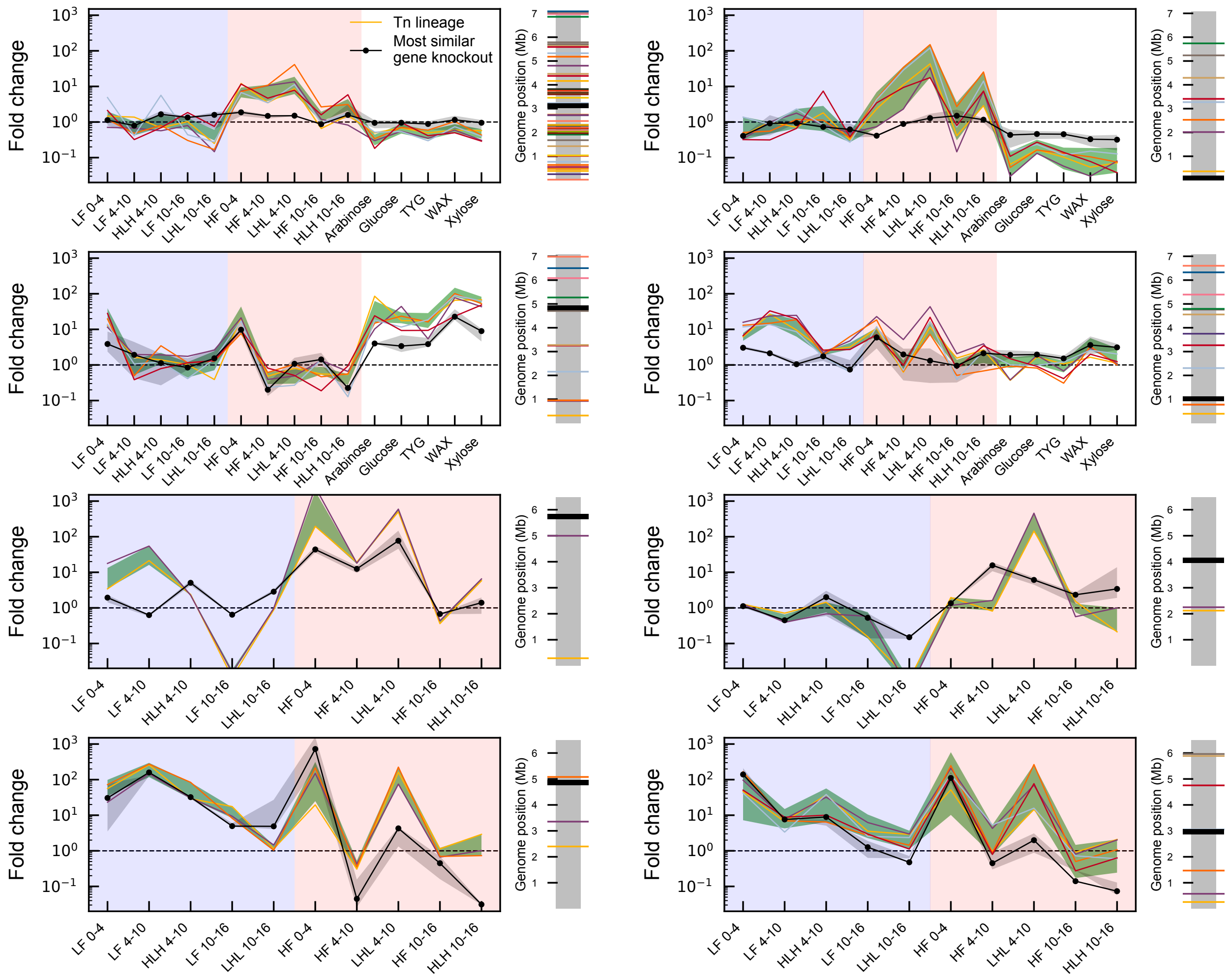

Supplementary Figure 15: Additional examples of lineages that are dissimilar to any gene knockout. Analogous versions of Fig. 4D for lineages in *Bc* (top 2 rows), *Bo* (third row), and *Bt-7330* (bottom row) that are similar to each other (colored curves and green band; Methods) but deviate from the most similar gene knockout in their respective libraries (black). For clusters with at least (fewer) than 10 lineages, green bands are IQR (full range). Lineage fitness profiling clustering in the *Bo* and *Bt-7330* libraries was performed with the same parameters as *Bc* (Methods).

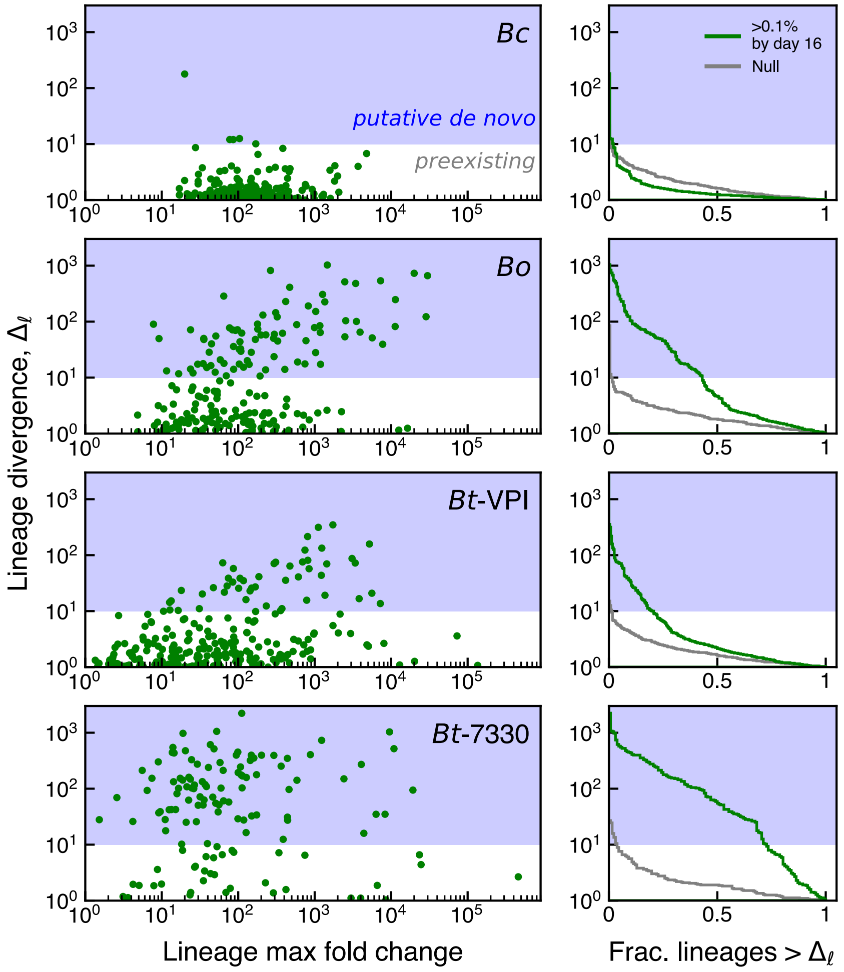

Supplementary Figure 16: Evidence for adaptive *de novo* mutations from the variability across mice. Left: lineage divergence as defined by Eq. (20) (a measure of the variability of a lineage across 5 HF/HS mice, Methods) as a function of the maximum fold change of that lineage in all mice from the same diet. Dozens of lineages that reached >0.1% frequency by day 16 in at least one mouse exhibited large divergences (>10, blue shaded region) at this time point, suggestive of putative *de novo* mutations. **Right**: survival function (green curve) of the divergence metric for the lineages in the left plot, as in Fig. 5B. For comparison, the survival function of divergences for a noise-matched null set of lineages is shown in grey.

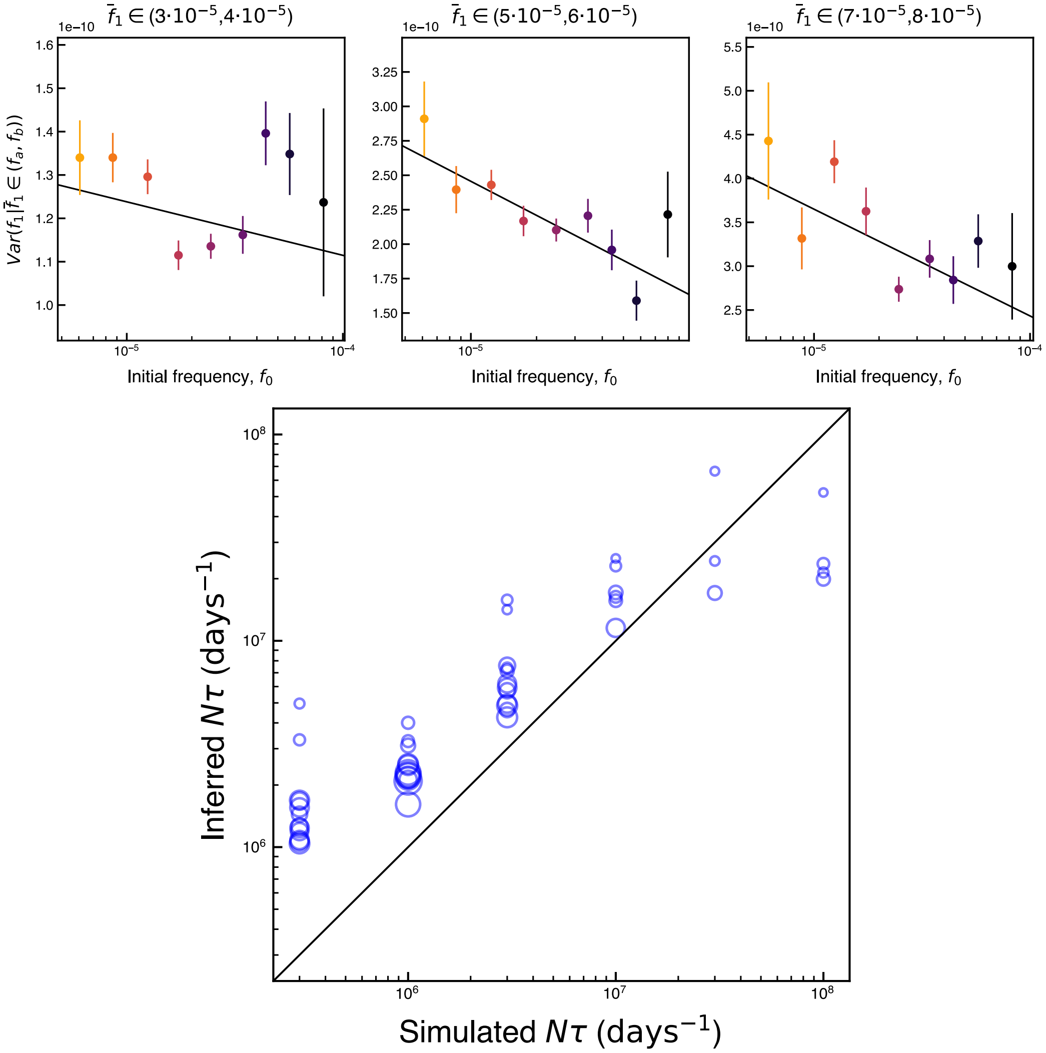

Supplementary Figure 17: Rates of genetic drift inferred from simulated data. Lineage dynamics over the first 4 days of colonization were simulated across 7 replicates for a given value of $N_e\tau_e$, using the empirical fitnesses estimated from the 7 *Bc* populations in the LF/HPP-fed mice (Methods). **Top panels:** analogous versions of Fig. 5D for three representative final frequency $\overline{f}_1$ windows when $N\tau = 3 \cdot 10^7$ days. **Bottom panel:** Inferred values of $N_e\tau_e$ using the algorithm described in the Methods. Each circle represents the inferred $N_e\tau_e$ from a single final frequency range, sized inversely to the relative uncertainty $|\sigma_{N\tau}/\hat{N\tau}|$. As in Fig. 5E, only regressions with $|\sigma_{N\tau}/\hat{N\tau}| < 1$, and effective sequencing noise uncertainty $|\sigma_{D_{\text{eff}}}/\hat{D}_{\text{eff}}| < 0.25$, are shown.

Supplementary References

References

[1] Good, B. H. & Desai, M. M. Fluctuations in fitness distributions and the effects of weak linked selection on sequence evolution. *Theoretical population biology* **85**, 86–102 (2013).

[2] Nguyen Ba, A. N. *et al.* High-resolution lineage tracking reveals travelling wave of adaptation in laboratory yeast. *Nature* **575**, 494–499 (2019).

[3] Levy, S. F. *et al.* Quantitative evolutionary dynamics using high-resolution lineage tracking. *Nature* **519**, 181–186 (2015).

[4] Venkataram, S. *et al.* Development of a Comprehensive Genotype-to-Fitness Map of Adaptation-Driving Mutations in Yeast. *Cell* **166**, 1585–1596.e22 (2016).

[5] Vasquez, K. S. *et al.* Quantifying rapid bacterial evolution and transmission within the mouse intestine. *Cell Host & Microbe* **29**, 1454–1468.e4 (2021).

[6] Venkataram, S., Kuo, H.-Y., Hom, E. F. Y. & Kryazhimskiy, S. Mutualism-enhancing mutations dominate early adaptation in a two-species microbial community. *Nature Ecology & Evolution* 1–12 (2023).

[7] Jiang, X. *et al.* Invertible promoters mediate bacterial phase variation, antibiotic resistance, and host adaptation in the gut. *Science* **363**, 181–187 (2019).

[8] Porter, N. T. *et al.* Phase-variable capsular polysaccharides and lipoproteins modify bacteriophage susceptibility in bacteroides thetaiotaomicron. *Nature Microbiology* **5**, 1170–1181 (2020).

[9] Shepherd, E. S., DeLoache, W. C., Pruss, K. M., Whitaker, W. R. & Sonnenburg, J. L. An exclusive metabolic niche enables strain engraftment in the gut microbiota. *Nature* **557**, 434–438 (2018).

[10] Zhao, S. *et al.* Adaptive Evolution within Gut Microbiomes of Healthy People. *Cell Host & Microbe* **25**, 656–667.e8 (2019).

[11] Lau, J. T. *et al.* Capturing the diversity of the human gut microbiota through culture-enriched molecular profiling. *Genome medicine* **8**, 1–10 (2016).

[12] Pryszlak, A. *et al.* Enrichment of gut microbiome strains for cultivation-free genome sequencing using droplet microfluidics. *Cell Reports Methods* **2**, 100137 (2022).

[13] Umkehrer, C. *et al.* Isolating live cell clones from barcoded populations using crispra-inducible reporters. *Nature Biotechnology* **39**, 174–178 (2021).

[14] Tenaillon, O. *et al.* The molecular diversity of adaptive convergence. *Science* **335**, 457–461 (2012).

[15] Price, M. N. *et al.* Mutant phenotypes for thousands of bacterial genes of unknown function. *Nature* **557**, 503–509 (2018).

[16] Jerison, E. R., Nguyen Ba, A. N., Desai, M. M. & Kryazhimskiy, S. Chance and necessity in the pleiotropic consequences of adaptation for budding yeast. *Nature Ecology & Evolution* **4**, 601–611 (2020).

[17] Kinsler, G., Geiler-Samerotte, K. & Petrov, D. A. Fitness variation across subtle environmental perturbations reveals local modularity and global pleiotropy of adaptation. *eLife* **9**, e61271 (2020).

[18] De Visser, J. A. G. & Krug, J. Empirical fitness landscapes and the predictability of evolution. *Nature Reviews Genetics* **15**, 480–490 (2014).

[19] Liu, H. *et al.* Functional genetics of human gut commensal Bacteroides thetaiotaomicron reveals metabolic requirements for growth across environments. *Cell Reports* **34**, 108789 (2021).

[20] Krimbas, C. B. & Tsakas, S. The genetics of dacus oleae. v. changes of esterase polymorphism in a natural population following insecticide control-selection or drift? *Evolution* **25**, 454–460 (1971).

[21] Nei, M. & Tajima, F. Genetic drift and estimation of effective population size. *Genetics* **98**, 625–640 (1981).

[22] Pollak, E. A New Method for Estimating the Effective Population Size from Allele Frequency Changes. *Genetics* **104**, 531–548 (1983).

[23] Feder, A. F., Kryazhimskiy, S. & Plotkin, J. B. Identifying signatures of selection in genetic time series. *Genetics* **196**, 509–522 (2014).

[24] Abel, S. *et al.* Sequence tag–based analysis of microbial population dynamics. *Nature methods* **12**, 223–226 (2015).

[25] Ascensao, J. A., Wetmore, K. M., Good, B. H., Arkin, A. P. & Hallatschek, O. Quantifying the local adaptive landscape of a nascent bacterial community. *Nature Communications* **14**, 248 (2023).

[26] Hullahalli, K., Pritchard, J. R. & Waldor, M. K. Refined quantification of infection bottlenecks and pathogen dissemination with stampr. *MSystems* **6**, e00887–21 (2021).

[27] Wu, M. *et al.* Genetic determinants of in vivo fitness and diet responsiveness in multiple human gut *Bacteroides*. *Science* **350**, aac5992 (2015).
