# Supplementary material for: Quantifying the adaptive landscape of commensal gut bacteria using high-resolution lineage tracking
Source: Nat Commun. 2024 Feb 21;15:1605. doi: 10.1038/s41467-024-45792-0 (PMC10881964; doi:10.1038/s41467-024-45792-0)
Supplement: Supplementary file 3 — Description of Additional Supplementary Files [file 41467_2024_45792_MOESM3_ESM.pdf]

Supplementary Data Files

Supplementary Data 1. List of samples and metadata used in this study.

Supplementary Code 1. Analysis code and figure generation scripts.
