# Supplementary figures and images for: Quantifying the adaptive landscape of commensal gut bacteria using high-resolution lineage tracking
Source: Nat Commun. 2024 Feb 21;15:1605. doi: 10.1038/s41467-024-45792-0 (PMC10881964; doi:10.1038/s41467-024-45792-0)

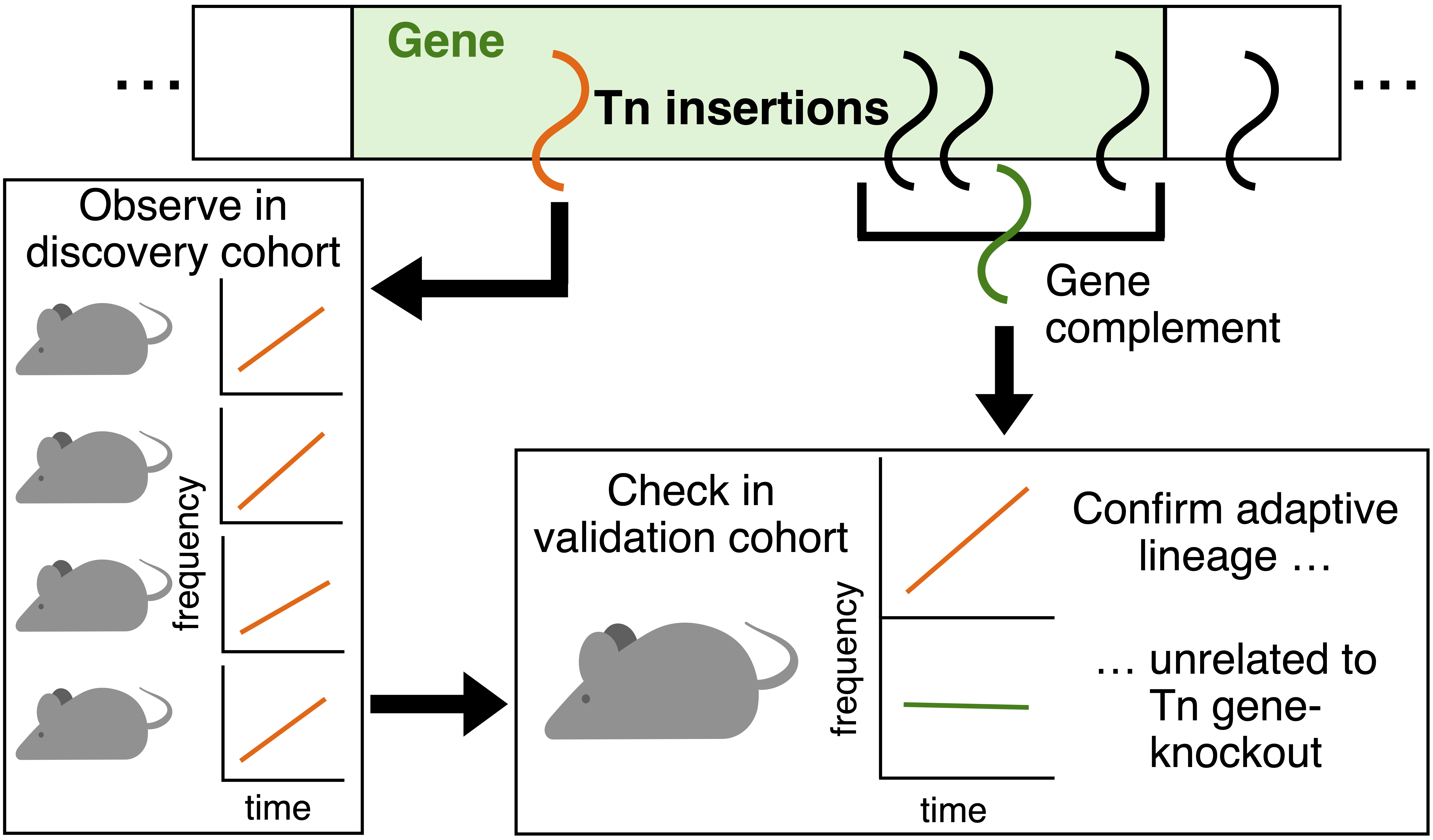

Supplement: Supplementary file 5 — Supplementary Code 1 [file 41467_2024_45792_MOESM5_ESM.zip › tnseq_adaptation_natcomm_fin/plots/fig2_schematic.png]

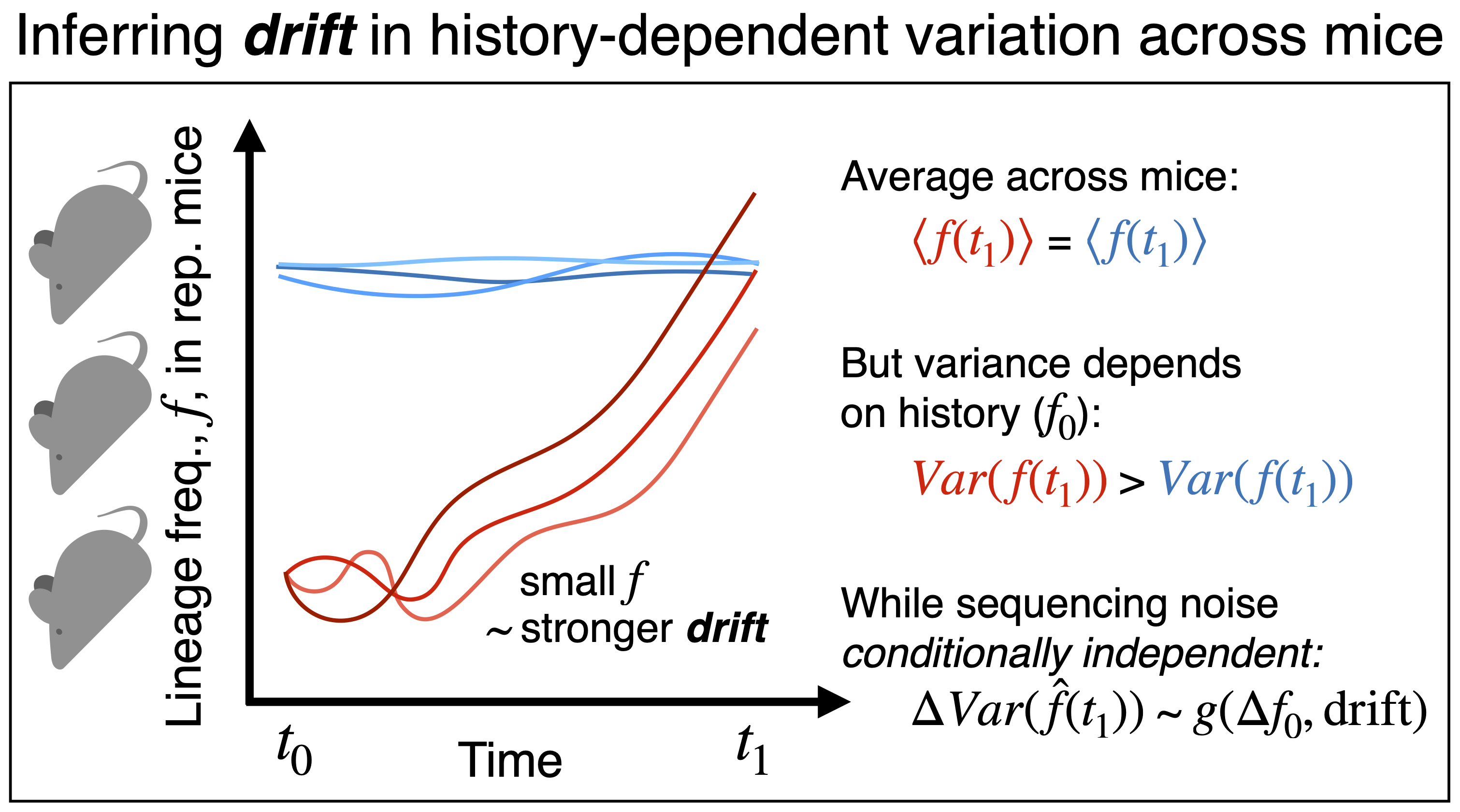

Supplement: Supplementary file 5 — Supplementary Code 1 [file 41467_2024_45792_MOESM5_ESM.zip › tnseq_adaptation_natcomm_fin/plots/fig5_drift_schematic.png]

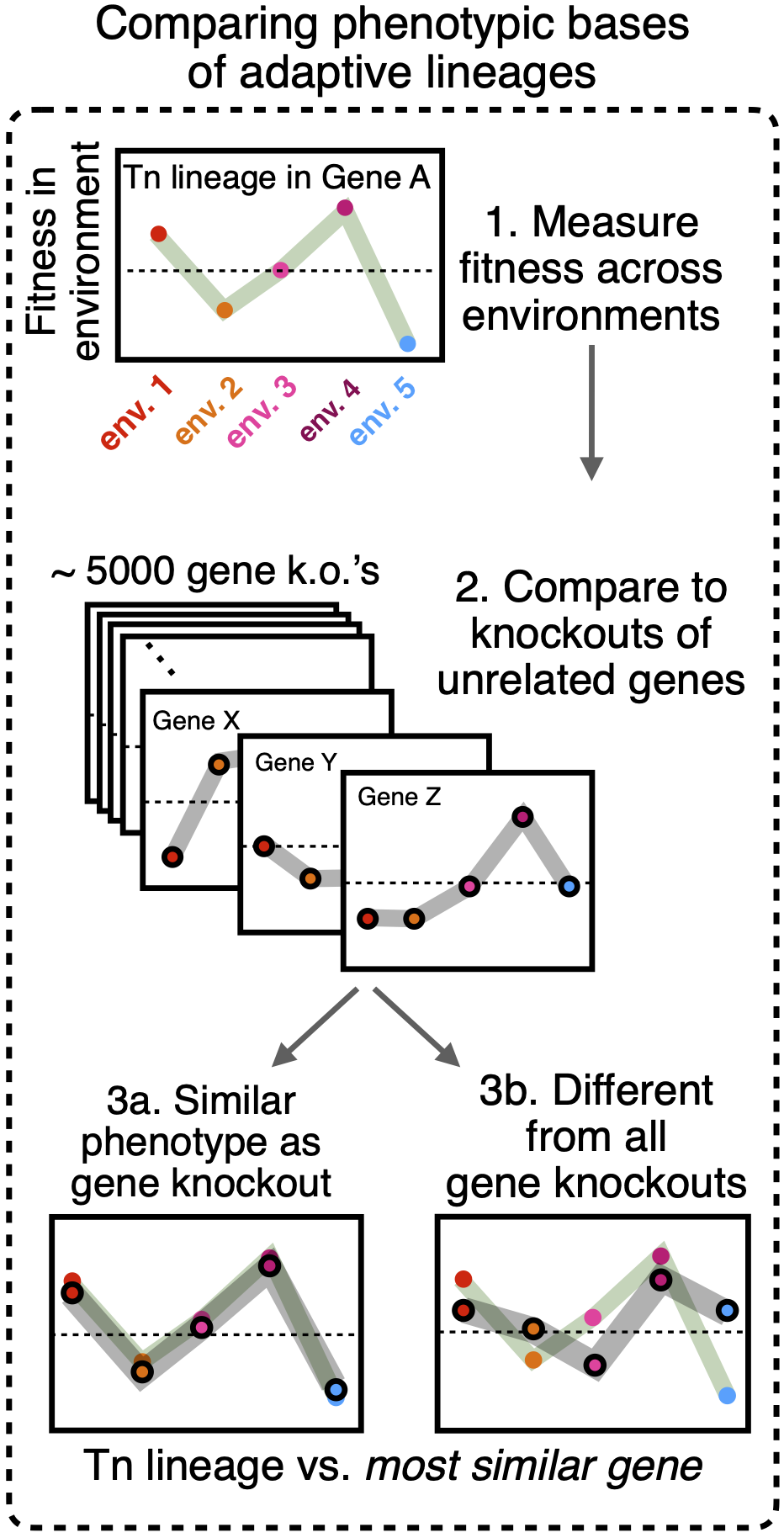

Supplement: Supplementary file 5 — Supplementary Code 1 [file 41467_2024_45792_MOESM5_ESM.zip › tnseq_adaptation_natcomm_fin/plots/fig4_schematic.png]

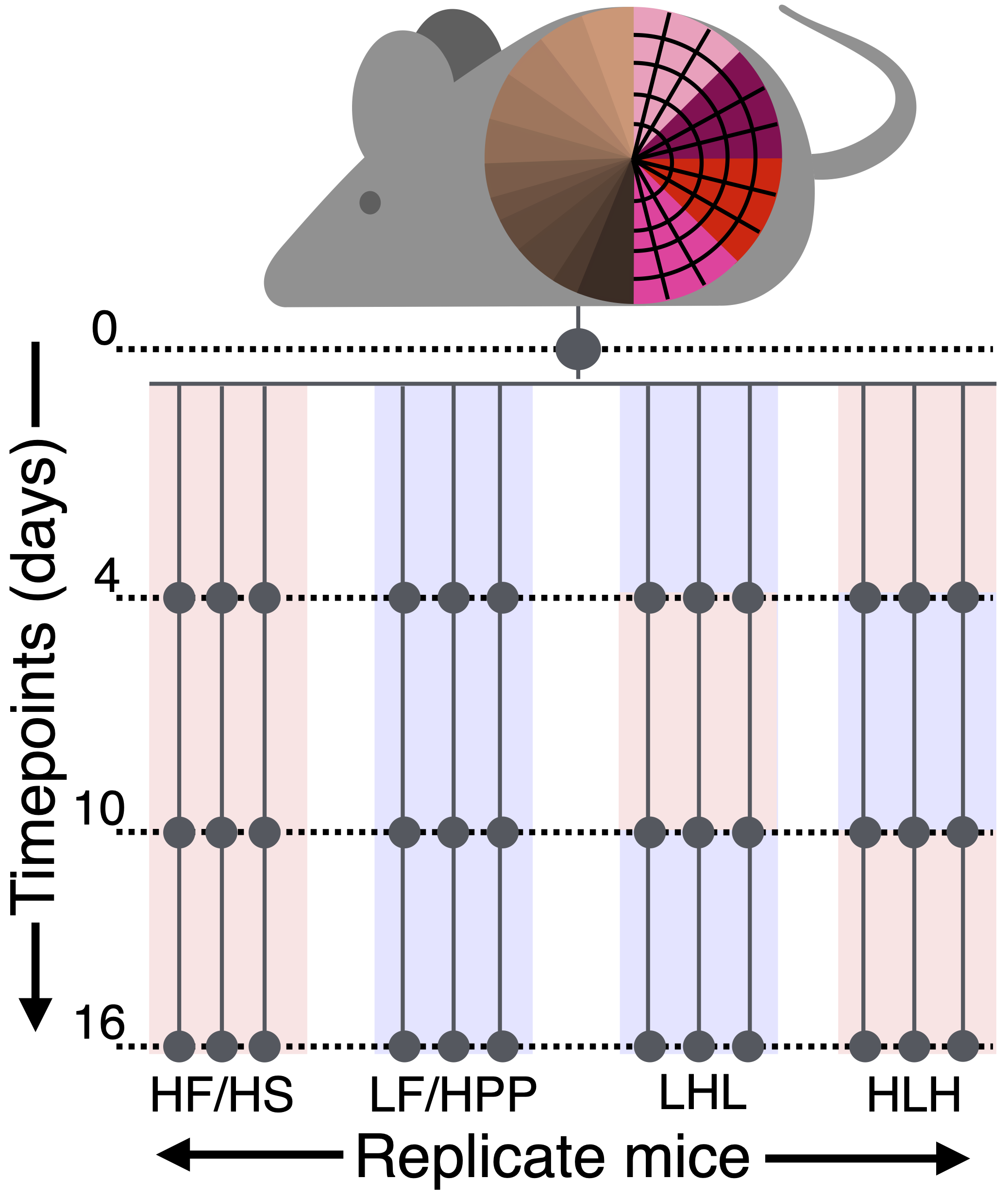

Supplement: Supplementary file 5 — Supplementary Code 1 [file 41467_2024_45792_MOESM5_ESM.zip › tnseq_adaptation_natcomm_fin/plots/lineage_schematic.png]
