# Supplementary material for: Quantifying the adaptive landscape of commensal gut bacteria using high-resolution lineage tracking
Source: Nat Commun. 2024 Feb 21;15:1605. doi: 10.1038/s41467-024-45792-0 (PMC10881964; doi:10.1038/s41467-024-45792-0)
Supplement: Supplementary file 6 — Reporting Summary [file 41467_2024_45792_MOESM6_ESM.pdf]

nature portfolio

Corresponding author(s): Benjamin H. Good

Last updated by author(s): Jan 29, 2024

Reporting Summary

Nature Portfolio wishes to improve the reproducibility of the work that we publish. This form provides structure for consistency and transparency in reporting. For further information on Nature Portfolio policies, see our Editorial Policies and the Editorial Policy Checklist.

Statistics

For all statistical analyses, confirm that the following items are present in the figure legend, table legend, main text, or Methods section.

n/a	Confirmed	
☐	☒	The exact sample size (*n*) for each experimental group/condition, given as a discrete number and unit of measurement
☐	☒	A statement on whether measurements were taken from distinct samples or whether the same sample was measured repeatedly
☐	☒	The statistical test(s) used AND whether they are one- or two-sided<br>*Only common tests should be described solely by name; describe more complex techniques in the Methods section.*
☒	☐	A description of all covariates tested
☐	☒	A description of any assumptions or corrections, such as tests of normality and adjustment for multiple comparisons
☐	☒	A full description of the statistical parameters including central tendency (e.g. means) or other basic estimates (e.g. regression coefficient) AND variation (e.g. standard deviation) or associated estimates of uncertainty (e.g. confidence intervals)
☐	☒	For null hypothesis testing, the test statistic (e.g. *F*, *t*, *r*) with confidence intervals, effect sizes, degrees of freedom and *P* value noted<br>*Give P values as exact values whenever suitable.*
☒	☐	For Bayesian analysis, information on the choice of priors and Markov chain Monte Carlo settings
☒	☐	For hierarchical and complex designs, identification of the appropriate level for tests and full reporting of outcomes
☐	☒	Estimates of effect sizes (e.g. Cohen's *d*, Pearson's *r*), indicating how they were calculated

Our web collection on statistics for biologists contains articles on many of the points above.

Software and code

Policy information about availability of computer code

Data collection	N/A (no new data was collected as part of this study).
Data analysis	This study utilized custom scripts deposited at the associated Github repository (https://github.com/bgoodlab/tnseq_adaptation) which was also provided in the supplemental code file.

For manuscripts utilizing custom algorithms or software that are central to the research but not yet described in published literature, software must be made available to editors and reviewers. We strongly encourage code deposition in a community repository (e.g. GitHub). See the Nature Portfolio guidelines for submitting code & software for further information.

Data

Policy information about availability of data

All manuscripts must include a data availability statement. This statement should provide the following information, where applicable:

- Accession codes, unique identifiers, or web links for publicly available datasets
- A description of any restrictions on data availability
- For clinical datasets or third party data, please ensure that the statement adheres to our policy

The full list of samples and technical replicates is provided in Supplementary Data 1.
Raw sequencing reads from the InSeq experiments were downloaded from the European Nucleotide Archive (accession no. PRJEB9434).
Raw sequencing reads from the input libraries were obtained from the authors of the original study (10.1126/science.aac5992). (Jeff Gordon, personal

communication)
Reference genomes for each of the 4 Bacteroides strains were obtained from National Center for Biotechnology Information (accession no. PRJNA289334).

Research involving human participants, their data, or biological material

Policy information about studies with human participants or human data. See also policy information about sex, gender (identity/presentation), and sexual orientation and race, ethnicity and racism.

Reporting on sex and gender	N/A
Reporting on race, ethnicity, or other socially relevant groupings	N/A
Population characteristics	N/A
Recruitment	N/A
Ethics oversight	N/A

Note that full information on the approval of the study protocol must also be provided in the manuscript.

Field-specific reporting

Please select the one below that is the best fit for your research. If you are not sure, read the appropriate sections before making your selection.

- [x] Life sciences
- [ ] Behavioural & social sciences
- [ ] Ecological, evolutionary & environmental sciences

For a reference copy of the document with all sections, see nature.com/documents/nr-reporting-summary-flat.pdf

Life sciences study design

All studies must disclose on these points even when the disclosure is negative.

Sample size	This work re-analyzes previously published data; for more information see the original paper (https://www.science.org/doi/full/10.1126/science.aac5992).<br>For each diet treatment (HF/HS, LF/HPP, HLH, LHL), 5 singly-housed gnotobiotic mice were gavaged with the defined 15-member community (including the 4 InSeq populations). Fecal pellets were collected from each mouse at regular intervals post-gavage (days 4, 10, 16).
Data exclusions	4 mice without InSeq sequencing at all 3 sampled time points (days 4, 10, 16) were excluded from statistical analysis.<br>No mice replicates with all 3 sampled time points were excluded from analysis.
Replication	As in the original paper, individually housed mice were treated as independent biological replicates for statistical purposes. No further replication (re-creation of InSeq libraries, new mouse experiments) was carried out.
Randomization	As described in the Supplementary Information of the original paper (see above), prior to microbiota colonization, 10-12 week male C57BL/6J were raised identically in germ-free conditions. Mice differed only in their diets, and no non-random basis for assigning mice to different diets is reported in the original study.
Blinding	Blinding was not relevant in the original or present studies, as analysis of InSeq libraries was independent of any (subjective) physiological or behavioral study of the mice.

Reporting for specific materials, systems and methods

We require information from authors about some types of materials, experimental systems and methods used in many studies. Here, indicate whether each material, system or method listed is relevant to your study. If you are not sure if a list item applies to your research, read the appropriate section before selecting a response.

Materials & experimental systems

n/a	Involved in the study	
x		Antibodies
x		Eukaryotic cell lines
x		Palaeontology and archaeology
x		Animals and other organisms
x		Clinical data
x		Dual use research of concern
x		Plants

Methods

n/a	Involved in the study	
x		ChIP-seq
x		Flow cytometry
x		MRI-based neuroimaging

Plants

Seed stocks	N/A
Novel plant genotypes	N/A
Authentication	N/A
